# Linking Subclinical Autistic Traits and Perceptual Category Learning

**DOI:** 10.1111/ejn.70000

**Published:** 2025-02-17

**Authors:** Claire V. Warren, Rebekka Baumert, Kira Diermann, Daniel Schöttle, Janine Bayer

**Affiliations:** ^1^ Department of Systems Neuroscience University Medical Center Hamburg‐Eppendorf Hamburg Germany; ^2^ Department of Clinical Psychology Helmut Schmidt University/University of the Federal Armed Forces Hamburg Hamburg Germany; ^3^ Department of Clinical Psychology and Psychotherapy Charlotte Fresenius University of Psychology Hamburg Germany; ^4^ Department of Psychiatry and Psychotherapy University Medical Center Hamburg‐Eppendorf Hamburg Germany

**Keywords:** autism, Bayesian computational modelling, dot‐pattern paradigm, fMRI, prototype abstraction

## Abstract

Autism spectrum condition is a neurodevelopmental condition with difficulties in social interaction, communication and repetitive behaviours. Autistic individuals often exhibit difficulties in non‐social cognitive processing, such as grouping items into meaningful categories based on their holistic visual appearance. Underlying mechanisms might be a deficit in abstracting a category's central tendency (i.e., the prototype) or more general atypicalities in visual category learning processes. Milder autistic traits often also extend to a broader autism phenotype in neurotypical individuals. Our study compared adult neurotypical individuals with high or low autistic traits on behavioural performances and neural correlates measured by event‐related functional magnetic resonance imaging (fMRI) during a single‐category perceptual categorization task, based on the well‐known dot‐pattern paradigm. Bayesian computational modelling was used to investigate links between autistic traits and representing category knowledge by the prototype or memorizing single exemplars. We found that a high degree of autistic traits was linked to worse accuracy for endorsing category members. Autistic trait groups also differed in neural correlates during the training phase related to visual processing in occipital regions, decision‐making in midfrontal regions and the posterior cingulate, and feedback processing in the posterior cingulate and the ventral striatum. Model‐based analyses did not support deficits in prototype abstraction but yielded a link between autistic traits and stricter decision policies. In sum, we found no relationship between high autistic traits and difficulties with the prototype strategy but more general atypicalities in visual category learning processes, namely, visual processing, decision‐making and feedback processing.

## Graphical abstract

Current theories of autism propose a tendency towards a detail‐oriented cognitive style of information processing, resulting in atypicalities during ‘perceptual categorization’. Here, we utilized a combined behavioural, Bayesian modelling and imaging approach to examine visual category learning processes in an adult neurotypical population with varying degrees of autistic traits. Individuals with a high degree of autistic traits showed worse accuracy for endorsing category members; though in contrast to our hypothesis, we found no relationship between high autistic traits and difficulties with the prototype strategy, that is, the ability to abstract a category's central tendency. However, we report a link between high autistic traits and more general atypicalities in visual category learning processes, namely, visual processing, decision‐making and feedback processing.

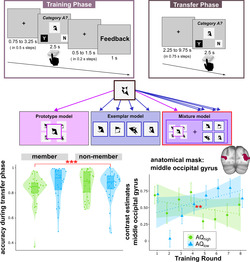

**Abbreviations**
ACC: anterior cingulate cortex
AIC: Akaike information criterion
AQ: Autism Quotient
BNA: Brainnetome Atlas
DIC: deviance information criterion
DL: distortion level
EQ: Empathy Quotient
(f)MRI: (functional) magnetic resonance imaging
FUS: fusiform gyrus
GLM: general linear model
HIPPEA: High, Inflexible Precision of Prediction Errors in Autism
IPS: intraparietal sulcus
ITG: inferior temporal gyrus
NEO‐FFI: NEO Five‐Factor Inventory
OCC: occipital cortex
PCC: posterior cingulate cortex
ROI: region of interest
*SD*
: standard deviation
SQ: Systemizing Quotient
VIFs: variance inflation factors

## 1 Introduction

Autism spectrum condition (ASC) refers to a characteristic profile of difficulties in social interaction, communication and repetitive behaviours (Ingersoll and Wainer 2014). Although classified as a neurodevelopmental disorder, autistic traits are also thought to be continuously prevalent at lower levels in the general neurotypical population as a broader autism phenotype (BAP) (Ingersoll and Wainer 2014; Landry and Chouinard 2016). Beyond the core autistic symptoms, individuals with autistic traits in ASC also exhibit atypicalities in non‐social cognitive processing, such as difficulties in the grouping of items into meaningful categories based on their holistic visual appearance (Mercado et al. 2020; Vanpaemel and Bayer 2021; Wimmer et al. 2023). This cognitive skill, also known as ‘perceptual categorization’, has high relevance to everyday learning and has even been discussed as an antecedent to social difficulties (Church et al. 2010; Mercado et al. 2020). However, it remains unclear exactly which mechanisms drive categorization difficulties and whether they also relate to the BAP in neurotypical individuals. The assessment of the link between cognitive processing and autistic traits in neurotypical samples has delivered valuable insight into potentially shared cognitive foundations of ASC and the BAP, being less confounded by methodological problems arising from studying clinical populations (e.g., the presence of comorbidities and educational differences). Additionally, studying this link in neurotypical individuals enhances our understanding of how personality traits relate to cognitive processes more broadly, independent of clinical diagnoses. The present article therefore examines autistic trait effects in a neurotypical sample on the different processes involved in category learning and representational strategies such as abstracting the category's central tendency (i.e., the prototype). Understanding autistic traits on the BAP may have implications for both the general population and ASC (Sasson and Bottema‐Beutel 2022).

Autism‐specific impairments have been reported predominantly for tasks akin to the single‐category dot‐pattern paradigm (Posner, Goldsmith, and Welton 1967; Vanpaemel and Bayer 2021), where abstract patterns must be grouped into category members and non‐members based on their holistic visual similarity (Church et al. 2010; Froehlich et al. 2012; Gastgeb et al. 2012; Klinger and Dawson 2001). Autism‐specific difficulties in these tasks are reflected in slower learning (Schipul and Just 2016; Vladusich et al. 2010), as well as lower accuracy rates (Church et al. 2010; Froehlich et al. 2012; Gastgeb et al. 2012; Klinger and Dawson 2001).

Similarity‐based category learning is supported by multiple cognitive processes (Seger and Miller 2010), for which autism‐specific atypicalities have been reported. The first of these processes is the visual processing of the stimuli, which relies on a broad network of lower and higher occipital regions (Lockhofen and Mulert 2021). Autism‐specific atypicalities in visual processing include an increased tendency towards detail‐focused visual processing (Bertone et al. 2005; Chung and Son 2020; Shah and Frith 1983), enhanced recruitment of lower visual regions (Kana et al. 2013; Ring et al. 1999) and structural alterations in visual cortices (Nickl‐Jockschat et al. 2012). A second process involved in similarity‐based category learning is perceptual decision‐making, which is mediated by a network of prefrontal regions and subcortical regions such as the anterior insula or the posterior cingulate cortex (PCC) (Keuken et al. 2014). In this subprocess, individuals must compare a to‐be‐categorized item to category representations and decide whether the items are sufficiently similar to justify endorsement as a category member. Autism‐specific atypicalities in this process are, for instance, more conservative response criteria, lower choice consistencies, as well as an atypical cytoarchitecture and neurochemistry in the PCC (Jassim et al. 2022; Oblak et al. 2011; Oblak, Gibbs, and Blatt 2011; Pirrone et al. 2017, 2020). Finally, category learning tasks often involve a training phase in which trial‐wise feedback is given, usually being mediated by a network of prefrontal and subcortical regions such as the ventral striatum (VS), and introducing a reward and punishment component to the task (Seger and Miller 2010). It is known that autistic individuals can show alterations in non‐social reward processing such as lowered striatal and increased prefrontal activations (Kohls et al. 2013; Scott‐Van Zeeland et al. 2010; Solomon et al. 2015; van Noordt et al. 2017) and sometimes perform better without negative feedback (Broadbent and Stokes 2013), though the extent of such alterations is often gender‐specific (Barman et al. 2015; Lawrence et al. 2020). In sum, known autistic atypicalities in visual processing, decision‐making and feedback processing may contribute to categorization difficulties.

Although the above‐mentioned processes for visual categorization based on similarity are universal, at least two individually distinct strategies can be used to form category representations (Blank and Bayer 2022; Bowman, Iwashita, and Zeithamova 2020; Bowman and Zeithamova 2018). The exemplar strategy requires category knowledge to be represented by memorizing individual instances of a category, whereas the prototype strategy requires similarities within a category to be identified and dissimilarities to be ignored in order to abstract its central tendency (Blank and Bayer 2022; Bowman, Iwashita, and Zeithamova 2020; Bowman and Zeithamova 2018; Medin and Schaffer 1978; Nosofsky 1988; Smith and Minda 2002). It has been proposed that individuals with autism exhibit specific difficulties in prototype abstraction, supported by the findings of a lower fit of a prototype model along with a reduced sensitivity to the prototype in autistic children (Church et al. 2010). Such difficulties could lead to worse categorization performances when the memory‐based exemplar strategy exceeds memory capacity, though this has not yet been examined.

The idea of autism‐specific difficulties in prototype abstraction is particularly appealing because it fits well with current theories of cognition in autism (Bölte et al. 2007; Brosnan et al. 2004; Happe and Frith 2006). Some cognitive theories of autism, for example, the weak central coherence (WCC) theory (Happe and Frith 2006) and the enhanced perceptual functioning (EPF) theory (Mottron et al. 2006; Van Eylen et al. 2018), assume that autists tend to focus on details, that is, local processing, a tendency that has also been observed in the BAP (Almeida et al. 2013; Bölte et al. 2007; Cribb et al. 2016). This may come at the expense of understanding the global meaning, which in turn could lead to difficulties in prototype abstraction. Newer cognitive theories such as Bayesian or predictive coding of autism provide more mechanistic explanations as to why individuals with autism might have difficulties distinguishing between category‐relevant and category‐irrelevant stimulus features. For instance, Bayesian approaches assume that the detail‐focused cognitive style can be accounted for by a bottom‐up processing style, due to a deficit in the integration of prior knowledge with incoming sensory input (e.g., hypo‐prior theory) (Pellicano and Burr 2012; Haker, Schneebeli, and Stephan 2016). Predictive coding approaches assume an inflexibly high weighting of data‐driven prediction errors (e.g., High, Inflexible Precision of Prediction Errors in Autism [HIPPEA] theory) (Van de Cruys et al. 2014). Both a reduced shaping of incoming perceptual input by the mental representation of a category (i.e., prior beliefs) and difficulties in ignoring category‐irrelevant differences between category members (i.e., prediction errors) could lead to categorization difficulties. It is worth noting that literature is currently inconclusive as to whether Bayesian and predictive coding approaches are considered as alternative theories or whether they represent complementary processes within the same explanatory framework (Angeletos Chrysaitis and Seriès 2023; Cannon et al. 2021; van Boxtel and Lu 2013).

In the current study, adult neurotypical individuals with high or low autistic traits (AQ_high_ vs. AQ_low_), as measured by the Autism Quotient (AQ) questionnaire, were compared on behavioural performances and neural correlates during a single‐category perceptual categorization task, based on the well‐known dot‐pattern paradigm (Posner, Goldsmith, and Welton 1967). The first aim of this study was to reproduce difficulties in perceptual categorization reported in autistic individuals compared to typically developing children (Church et al. 2010). The second aim was to uncover underlying mechanisms of perceptual category learning by relating autistic traits to neural correlates of visual processing, decision‐making and feedback processing. Supported by Bayesian computational modelling, the final aim was to link autistic traits with representing category knowledge either by the prototype or by exemplars and with neural prototype and exemplar representations.

Based on existing literature, we had the following hypotheses:

1. AQ_high_ individuals would show worse performances in the category learning task compared to AQ_low_ individuals.
2. Accuracy would be negatively correlated specifically with the attention to detail subscale of the AQ questionnaire.
3. Autistic trait groups would differ during the category learning task between neural correlates of visual processing, decision‐making and feedback processing.
4. AQ_high_ individuals would make less use of the prototype strategy compared to AQ_low_ individuals.
5. Neural prototype representations differ between autistic trait groups.

## 2 Materials and Methods

### 2.1 Sample Characteristics

A total of *N* = 72 healthy volunteers took part in the study. Volunteers were recruited via advertisement on a job board of the University of Hamburg. Inclusion criteria were being between 18 and 35 years old, fluent in the German language, having no history of mental or neurological disease, having no drug or alcohol abuse, and having no contraindications to MRI measurement (e.g., metal implants and pregnancy). All volunteers were required to have normal or, through contact lenses, corrected‐to‐normal vision. All gave signed informed consent according to the Declaration of Helsinki. Ethics approval was obtained from the Ethics Committee of the Hamburg Medical Association (PV5874). All participants filled out questionnaires and performed behavioural tasks (detailed below) on their first visit and underwent MRI scanning during category learning on their second visit.

Two volunteers did not complete the measurement due to discomfort in the scanner, one measurement was interrupted by technical problems, and one volunteer was measured with the wrong sequence settings. Datasets from these volunteers were excluded from all analyses. In addition, volunteers were excluded if they performed below 75% accuracy during the last training block of the category learning task and did not reach an average performance of 75% during the test phase (*n* = 5). The data from an additional volunteer were excluded because the fit of the guessing model did not show a meaningful difference to the fits of the prototype, exemplar and mixture models. Finally, data were removed from one participant who reached a sum score of 34 in the AQ (Baron‐Cohen et al. 2001), which was higher than 3 *SD* above the group mean and falls above the AQ's diagnostic cut‐off value of 32 (see Section 3.1).

In total, data from *N* = 61 volunteers were used for further analyses. Included volunteers were between 19 and 34 years old (*M* = 25.6, *SD* = 3.7 years). Of these, *n* = 30 of the volunteers were female and *n* = 8 were left‐handed. Volunteers had at least 12 years of education, with the majority being currently enrolled as university students (*n* = 38).

Please note that parts of the dataset have already been published in an article focusing on the localization of prototype and exemplar representations (Blank and Bayer 2022).

### 2.2 Questionnaires and Logical Reasoning Task

The 50‐item version of the AQ questionnaire was used to measure autistic traits (Baron‐Cohen et al. 2001). The AQ is a self‐report measure in which participants rate the personal relevance of statements concerning potential autistic traits (e.g., ‘I enjoy meeting new people’) according to a 4‐point Likert scale (*definitely agree* to *definitely disagree*). Participants can score a range of 0–50, with a higher score indicating more autistic traits. In addition to the sum score, scores for the five AQ subscales were calculated (i.e., ‘social skills’, ‘communication’, ‘attention to detail’, ‘attention switching’ and ‘imagination’). The AQ also shows high test–retest reliability, internal consistency, sensitivity and specificity in clinical as well as subclinical populations (Baron‐Cohen et al. 2001). Crucially for our examination of visual categorization, the AQ proved to be the most suitable due to a specific subscale measuring traits related to attention to detail. Ultimately, the AQ measures a wider breadth of social and non‐social subscales than, for example, the Social Responsiveness Scale (SRS) (Constantino 2021) and the Broad Autism Phenotype Questionnaire (BAPQ) (Hurley et al. 2007). The AQ also focuses on symptoms themselves as opposed to impairment (e.g., as in the SRS) and is the most widely used of all available scales (Ruzich et al. 2015).

To generate working hypotheses for future research, we complemented the characterization of personality traits using the Empathy Quotient (EQ) (Baron‐Cohen and Wheelwright 2004), the Systemizing Quotient (SQ) (Baron‐Cohen et al. 2003) and the NEO Five‐Factor Inventory (NEO‐FFI) (Borkenau and Ostendorf 1993). The EQ (range: 0–80) requires subjects to respond to 40 empathy statements (e.g., ‘It upsets me to see an animal in pain.’) on a 4‐point Likert scale. The SQ is a self‐report measure of analytical cognitive style (range: 0–80), in which participants use the same 4‐point Likert scale to rate statements concerning systemizing (e.g., ‘I prefer to read non‐fiction than fiction.’). Both the EQ and SQ questionnaires measure constructs that partially overlap with subscales of the AQ.

To control for potential differences in fluid intelligence between AQ groups, logical reasoning was assessed using the short version of the Figure Reasoning Test (FRT; [Daniels, Booth, and Horn 1962]). The FRT is a language‐free intelligence test where individuals have to identify the missing figure in a 3 × 3 matrix of geometric structures by selecting the correct option from six given alternatives, with a total of 45 tasks to complete.

### 2.3 Working Memory Task

We used a modified version of an *n*‐back task as a control measure, to address the possibility that differences in visual working memory performance between AQ groups might influence the hypothesized differences in perceptual category learning (Rabi and Minda 2017; Smith, Jackson, and Church 2019). The stimulus set consisted of 22 letters from the Hindi and Sanskrit alphabet. The specific stimulus set was chosen due to being relatively complex and novel to the participants, like the stimuli generated for the category learning task. Individuals were asked to hit the left mouse button when a stimulus occurred for the first time and the right mouse button when a stimulus was a repetition of the last (1‐back condition) or the penultimate stimulus (2‐back condition). A practice round with trial‐wise feedback ensured that individuals understood the task.

Individuals performed two blocks of the 1‐back and two blocks of the 2‐back condition in an alternating manner. Each block consisted of 27 stimuli with 10 targets. Each stimulus was presented for 1 s, followed by a fixation cross presented at a duration of 250 ms.

Working memory performance was measured by the rate of correct responses in the 1‐back condition minus the rate of correct responses in the 2‐back condition.

### 2.4 Dot‐Pattern Category Learning Task

**FIGURE 1:**
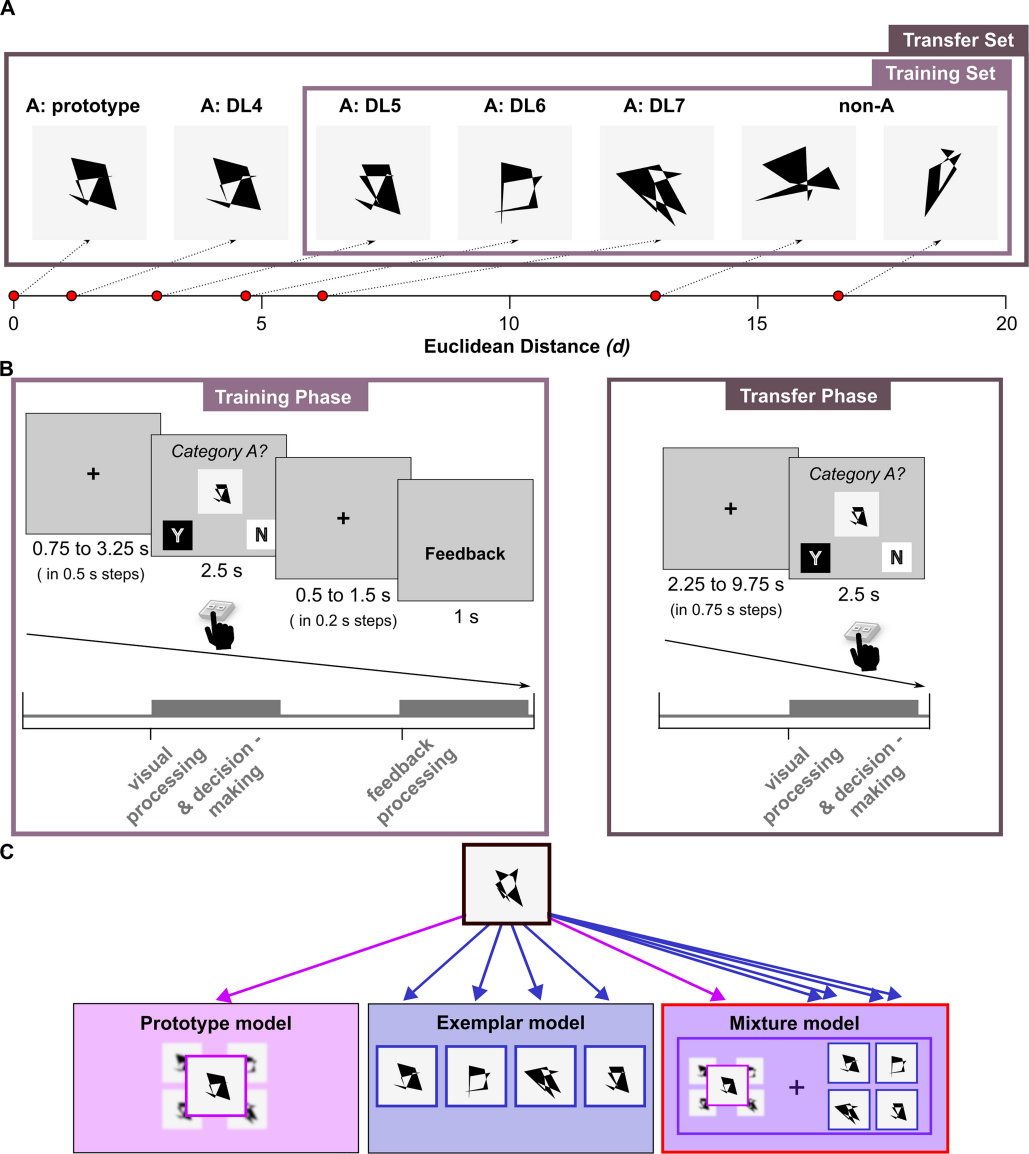
(A) Examples of stimuli from the training and transfer sets for each distortion level (DL), which were set to a visual angle of 4.22°. (B) Task design (upper row) along with fMRI events (bottom row) of universal category learning subprocesses. Please note that the event durations shown are based on the illustration of the task design and are not to scale. (C) Schematic illustration of how a categorized item is compared to the prototype (prototype model), exemplar (exemplar model) or both (mixture model). As the model type with the best fit, the mixture model box is surrounded by a red frame.

The current study employed a modified version of a classical A/not‐A dot‐pattern paradigm. The task consisted of a training phase in which participants learned to categorize stimuli via feedback, followed by a transfer phase to assess their ability to generalize this learning when no feedback is given and new stimuli are introduced. Dot patterns were created using a well‐established procedure originally published by Posner, Goldsmith, and Welton (1967). The full stimulus set consisted of polygons made from 41 patterns of 9 random static dots, with one being the category's prototype, 20 distortions of the prototype (i.e., category members) and 20 unrelated random patterns (i.e., non‐members). The post hoc calculation of Euclidean distance to the category prototype yielded a maximum of 6.22 for category members and a minimum of 12.93 for non‐members. The training set consisted of 8 medium distorted (i.e., Distortion Levels 5 [DL5] and 6 [DL6]) and 4 highly distorted (DL7) category members and 12 random patterns. The transfer set consisted of the prototype, the 24 stimuli from the training set, 8 novel distorted versions of the prototype (DL4, DL5, DL6 and DL7) and 8 novel random patterns. Example stimuli are shown in Figure 1A. Please refer to Blank and Bayer (2022) for a detailed description of the construction of the stimulus set.

Before entering the MRI machine, participants were told they would learn via trial and error whether abstract patterns belong to a certain category (‘A’) or not. They were instructed that only the overall similarity of stimuli was informative of category membership. Before training with the actual stimulus set, two familiarization rounds consisting of 24 trials with a different stimulus set ensured that volunteers understood the task.

The main categorization task consisted of a training phase and a transfer phase, both performed in the MRI (Figure 1B). In each training round, the same 12 category members and 12 non‐members were presented in pseudorandomized order. Please note that the prototype was not shown during the training phase. Each trial began with the presentation of a fixation cross with a jittered duration of 0.75–3.25 s. Next, one of the patterns was presented in the centre of the screen. The stimulus size in the visual field was set to a visual angle of 4.22°. Individuals had to indicate via button press within a time window of 2.5 s, whether they believed a pattern belonged to category ‘A’ or not, followed by a fixation cross with a duration of 0.5–1.5 s. Next, visual feedback was presented indicating whether the volunteers' choice was ‘correct’, ‘incorrect’ or ‘too late’. Total accuracy per round was shown after each training round. The training phase continued until completing at least eight rounds and four rounds with at least 75% correct or after the completion of all 14 training rounds. In each transfer round, all 24 stimuli from the training set plus 17 novel stimuli (*n* = 9 category members including the prototype and 8 non‐members) were presented in pseudorandomized order. As in the training phase, volunteers indicated suspected category membership via button press but without receiving any feedback.

### 2.5 Computational Modelling of Performances in the Category Learning Task

Single‐category prototype, exemplar and mixture Bayesian models (Medin, Altom, and Murphy 1984; Minda and Smith 2001; Nosofsky 1988; Posner and Keele 1968) (Figure 1C) were adopted to investigate individual prototype and exemplar strategy preferences as well as neural representations. We specifically employed Bayesian computational modelling because it provides a robust framework for comparing cognitive models such as prototype, exemplar and mixture models (Bartlema et al. 2014; Blank and Bayer 2022; Voorspoels et al. 2018). This approach allowed us to incorporate prior knowledge and manage model complexity. Additionally, using multiple Markov chains in Bayesian modelling enhances the reliability of convergence and ensures comprehensive exploration of the posterior distribution, reducing the risk of local minima and increasing the robustness of our results. This approach was particularly suited for our research questions, as it enabled us to explore individual differences in categorization strategies with greater precision and confidence.

A guessing model was estimated assuming endorsement probability to be at chance level, to identify volunteers whose performances could be better explained by guessing than by a specific representational strategy (Voorspoels et al. 2018). For model estimation on data from the first eight training rounds, a binary vector coding whether an item has been endorsed as a category member was submitted separately for each run to model estimation. For model estimation on data from the transfer phase, the endorsement variable was averaged across transfer rounds, and individual endorsement rate vectors were submitted to model estimation. For simplicity's sake, the formula below describes parameter calculation for the transfer phase.

All three model types define perceptual similarity (*s*) as an exponential decay function with a sensitivity parameter (*c*), quantifying the steepness of how perceptual similarity decays with distance (*d*). The prototype model postulates that the similarity of item *i* to Category A is represented by its similarity to the prototype *P* ($s_{\mathrm{iPA}}$; 1). The exemplar model postulates that the similarity of item *i* to Category A is represented by the summed similarity to training exemplars *j* ($s_{\text{iExA}}$; 2). The mixture model assumes that the similarity of item *i* to Category A is represented by both its similarity to the prototype *P* ($s_{\mathrm{iPA}}$; 1) and the summed similarity to training exemplars ($s_{\text{iExA}}$; 2).

1 Similarity of the *i*th item to prototype: $s_{\mathrm{iPA}}=e^{-cd_{\mathrm{iP}}}$
2 Similarity of the *i*th item to exemplars *j*: $s_{\text{iExA}}=\sum_{j\in A}e^{-{\mathrm{cd}}_{\mathrm{ij}}}$

Perceptual similarity (*s*) is derived using an exponential function in which physical distances are scaled by an individual sensitivity parameter (*c*) taken to the power of the Euler's number (1/2). Accordingly, physical distances farther away from the prototype or exemplars are down‐weighted, with the amount of down‐weighting being determined by individual sensitivity. The resulting estimates of perceptual similarity have been shown to closely match similarity ratings in previous studies (Posner, Goldsmith, and Welton 1967; Smith and Minda 2002). The basic version of the mixture model (‘MIX’) assumes only one common sensitivity parameter *c* for both the prototype and the exemplar process, whereas an extended version of the mixture model assumes separate sensitivity parameters for the two processes (‘MIX‐2c’).

In the restricted version of the models (‘PROTO’, ‘EX’ and ‘MIX’), the endorsement probabilities (*r*) are based on the similarity to Category A plus a criterion parameter (*k*). In detail, the endorsement probability estimation in prototype (${\text{rproto}}_{i})$ and exemplar models (${\mathrm{rex}}_{i}$) relies on either $s_{\mathrm{iPA}}$ (Formula 3) or $s_{\text{iExA}}$ (Formula 4). In the mixture models, ${\text{rproto}}_{i}$ and ${\mathrm{rex}}_{i}$ are weighted with a mixture parameter *β* and then summed (Formula 5). The mixture parameter varies between 0 and 1, with *β* = 0 meaning that decisions are only based on similarity to exemplars and *β* = 1 meaning that decisions are only based on similarity to the prototype. The guessing model assumes that all endorsement probabilities *r* are 0.5.

3 Endorsement probability of the *i*th item for prototype model: ${\text{rproto}}_{i}=\frac{s_{\mathrm{iPA}}}{s_{\mathrm{iPA}}+k}$
4 Endorsement probability of the *i*th item for exemplar model: ${\mathrm{rex}}_{i}=\frac{\;s_{\text{iExA}}}{\;s_{\text{iExA}}+k}$
5 Endorsement probability of the *i*th item for mixture model:
$${\text{rmix}}_{i}=\beta *{\text{rproto}}_{i}+\left(1-\beta \right)*{\mathrm{rex}}_{i}$$

The extended versions of the exemplar (‘EX‐*γ*’) and mixture models (‘MIX‐*γ*’ and ‘MIX‐2c‐*γ*’) contain an additional response scaling parameter (*γ*) (Navarro 2007; Vanpaemel 2016), which allows less (*γ* < 1) or more deterministic choices (*γ* > 1) than the restricted versions of the models.

6 Exemplar‐based endorsement probability of the *i*th item for EX‐*γ*, MIX‐*γ* and MIX‐2c‐*γ*: ${\mathrm{rex}}_{i}=\frac{s_{\text{iExA}}^{\gamma }}{s_{\text{iExA}}^{\gamma }+k^{\gamma }}$

Model fitting was performed using the package ‘R2jags’ (Su et al. 2015) under R Version 4.0.3 (R Core Team 2020), which implements Bayesian analysis in JAGS (Plummer 2003). All three models were fit to endorsement rates of the transfer phase individually for each volunteer. Uniform priors were used for sensitivity (*c* ~ uniform(0,5)), criterion (*k* ~ uniform(0,1)), mixture (*β* ~ uniform(0,1)) and response scaling parameters (*γ* ~ uniform(0,20)), representing beliefs before fitting the models to the data that *c* varies between 0 and 5, *k* between 0 and 1, *β* between 0 and 1, and *γ* between 0 and 20 with homogenous probabilities. Markov chain Monte Carlo (MCMC) algorithms provided by the JAGS software were used for parameter estimation. Starting values for parameter estimation were determined by first fitting the two models without initial value definition (4000 repetitions, burn‐in period of 500 repetitions, four chains) and then using the 2.5%, 25%, 50%, 75% and 97.5% quantiles of the posterior distributions (i.e., the updated beliefs after model fitting to the data) as initial values for the next modelling step. The final model estimation was performed using 20,000 repetitions, a burn‐in period of 2500 repetitions and four chains. To reduce autocorrelations, a thinning factor of 5 was used so that only every fifth sample from the posterior distribution was kept. Chain convergence diagnosis, that is, testing whether the simulated draws reached their stationary state, was performed by visual inspection and by calculating the potential scale reduction factor (Rhat) (Gelman et al. 2021). Model fits were measured by the deviance information criterion (DIC) (Spiegelhalter et al. 2002), which is a hierarchical modelling generalization of the Akaike information criterion (AIC) and is provided by the JAGS software as a default measure for Bayesian model fit. Chain convergence for the prototype model, fitted to data from the training phase of a task, required increasing the number of model estimation repetitions to 100,000. No chain convergence could be obtained for the MIX‐*γ* and MIX‐2c*‐γ* models estimated on data from the transfer phase, so that model fits and parameter estimates from these models were not used for further analyses. Posterior means of posterior distributions for the free parameters were used for subsequent analyses, including the generation of trial‐wise similarity vectors for each individual volunteer (Formulas 1 and 2).

Trial‐wise similarity estimates of category members were used as parametric modulators in fMRI analyses (Blank and Bayer 2022). Please note that *prototype* and *exemplar similarity vectors* for category members during the training phase were highly correlated (*M*_*r*_ = 0.701, *SD*_*r*_ = 0.164), so that the two similarity vectors were combined to a single parametric modulator (${\text{smix}}_{i}=\beta *{\text{sproto}}_{i}+\left(1-\beta \right)*{\mathrm{sex}}_{i}$; ‘mixture similarity vector’). This was not the case for the transfer phase (*M*_*r*_ = −0.256, *SD*_*r*_ = 0.065), allowing to investigate separate neural prototype and exemplar representations.

### 2.6 fMRI Acquisition

Event‐related functional MRI was performed on a 3‐T Siemens PRISMA scanner with a multiband gradient echo planar imaging T2*‐weighted sequence in 54 contiguous axial slices (2‐mm thickness, TR of 1.636 s, TE of 29 ms, flip angle of 70°, field of view of 224 × 224 and multiband factor of 2). For spatial normalization, a high‐resolution T1‐weighted structural MR image was acquired by using a 3D‐MPRAGE sequence (TR of 2300 ms, TE of 2.89 ms, flip angle of 9°, 1‐mm slices and field of view of 256 × 192; 240 slices).

Event‐related fMRI data were preprocessed and analysed using Statistical Parametric Mapping (SPM12; Wellcome Department of Imaging Neuroscience, London, UK) (Penny et al. 2011) run in Matlab R2014b. To prevent biases due to spin saturation, the first five functional images were discarded. All functional images were realigned and unwarped (as implemented in SPM12) to correct for susceptibility‐by‐movement artefacts. Individual structural T1 images were coregistered to functional images, segmented into grey and white matter and submitted to the ‘diffeomorphic anatomic registration through an exponentiated lie algebra algorithm’ (DARTEL) toolbox to create structural templates, individual flow fields, and subject‐specific grey matter, white matter and cerebrospinal fluid (CBF) masks. Flow fields were used for normalizing structural and functional images to MNI space. Functional images were smoothed with a full width at half maximum Gaussian kernel of 8 mm in all spatial directions. White matter and CBF masks were used to extract time series representing noise unrelated to the experimental paradigm. Principal components explaining at least 1% of variance were used as nuisance regressors in all first‐level models.

### 2.7 fMRI Analyses

Normalized functional images from the first eight rounds of the training phase and from all eight transfer rounds were submitted to separate general linear models as implemented in SPM using a mass univariate approach, in order to investigate whether autistic trait groups differ in neural correlates of universal category learning processes (i.e., visual processing, decision‐making and feedback processing) or neural prototype and exemplar representations. For all subject‐level GLMs, fMRI runs were concatenated, and a session constant was added.

Subject‐level GLMs investigating neural correlates of universal category learning processes during the training phase included separate regressors for each round. These included boxcar regressors for the time periods between stimulus onset and stimulus offset and visual feedback onset until visual feedback offset for correctly and incorrectly identified member and non‐member stimuli (factor *membership*). Regressors containing onsets of fixation crosses for each round were used as an explicit baseline. To explain additional variance, nuisance regressors were added as regressors of no interest. Subject‐level GLMs investigating neural correlates of universal categorization processes during the transfer phase were similar to GLMs for the training phase with the exception of collapsing rounds (factor *round*) and containing no feedback onset regressors.

Subject‐level GLMs investigating prototype and exemplar representations during the training and transfer phases contained an onset regressor for correctly categorized category members, modulated by the mixture similarity vector (i.e., training‐mixture GLM; see Section 2.5 for the formulas to derive similarity values), the prototype similarity vector or the exemplar similarity vector (i.e., transfer‐prototype and transfer‐exemplar GLMs). To explain additional variance, the following regressors of no interest were added: onsets for correctly classified non‐members, incorrectly categorized patterns, button presses and fixation crosses, and nuisance regressors.

To create individual contrast images representing relevant main effects of universal category learning processes, the stimulus regressors were contrasted against fixation cross onset vectors, and feedback regressors for correctly categorized stimuli (‘positive feedback’) were contrasted against those for incorrectly categorized stimuli (‘negative feedback’). Individual contrast images representing neural prototype and exemplar representations were generated by applying a contrast of 1 to the respective parametric modulators. Contrast images were submitted to two‐sample *t‐*tests with the between‐subject factor *group* (AQ_high_ vs. AQ_low_). Contrast estimates from all peaks surviving a threshold of *p* < 0.05, family‐wise error corrected for multiple comparisons on the entire scan volume and predefined regions of interest (ROIs) (collapsed by processing stage) were considered as significant. ROI‐based analyses were performed by submitting contrast estimates extracted from ROIs (Section 2.8) to robust linear mixed models (see Section 2.9). Results were considered significant when surviving a Bonferroni‐corrected threshold of *p* < 0.05, with multiple‐comparison corrections being based on the number of comparisons for each research question (e.g., number of visual processing ROIs; see Supporting Information S1: Table 1).

### 2.8 ROIs

ROI selection was informed by previous literature on visual processing in perceptual category learning tasks, perceptual decision‐making (Keuken et al. 2014), feedback processing (Kéri 2003; Kohls et al. 2013; Little et al. 2006; Peters et al. 2014; Scott‐Van Zeeland et al. 2010) and neural prototype/exemplar representations (Blank and Bayer 2022).

Drawing upon prior research into visual processing during perceptual category learning (Braunlich, Liu, and Seger 2017; Little et al. 2006; Reber, Stark, and Squire 1998), we have selected specific subregions of the posterior occipital cortex (OCC), the lateral OCC, the fusiform gyrus (FUS) and the inferior temporal gyrus (ITG) for our study. Building upon literature on perceptual decision‐making (Keuken et al. 2014), we chose particular subregions of the medial superior frontal gyrus (SFG), the middle frontal gyrus (MFG), the inferior frontal gyrus (IFG), the anterior insula, the ventromedial putamen and the PCC. Based on findings on feedback processing (Kéri 2003; Kohls et al. 2013; Little et al. 2006; Peters et al. 2014; Scott‐Van Zeeland et al. 2010), we selected subregions in the IFG, the SFG, the anterior cingulate cortex (ACC), the PCC, the VS and the dorsal striatum. To investigate prototype and exemplar representations (Blank and Bayer 2022), we selected subregions of the FUS, the lateral OCC, the posterior OCC and the frontal gyrus, which showed prototype and/or exemplar representations in previous univariate fMRI analyses (Blank and Bayer 2022). For additional exploratory analyses, we used subregions in the intraparietal sulcus (IPS) that showed prototype and exemplar representations in multivariate representational similarity analyses (Blank and Bayer 2022) and a correlation with decision bound in a similar category learning task (Braunlich, Liu, and Seger 2017).

All masks were created by using the Brainnetome Atlas (BNA) (Fan et al. 2016). Supporting Information S1: Table 1 lists the selected subregions with the corresponding BNA labels. Separate masks were used for the two hemispheres.

### 2.9 Robust Linear (Mixed) Models for Behavioural and ROI‐Based fMRI Analyses

Multiple comparison‐corrected ROI‐based analyses were conducted using a median split grouping variable based on sum scores in the AQ questionnaire to balance statistical power and control type I error (Baron‐Cohen et al. 2001; DeCoster, Gallucci, and Iselin 2011). This approach enabled a focused examination of complex interactions, such as those between category membership and training round (see below and Supporting Information S1: Section 2). Resulting groups were labelled AQ_low_ and AQ_high_ (factor *group*).

To investigate differences between groups in categorization performances, robust linear models were estimated using the ‘rlm()’ function from the ‘MASS’ package (Venables and Ripley 2013), and robust linear mixed models were estimated using the ‘rlmer()’ function from the ‘robustlmm’ package (Koller 2016) in R (R Core Team 2020) in order to minimize the impact of outlier values. In all models, only linear effects were used for result interpretation.

Before entering data into regression models, behavioural data were aggregated as follows: For regression models investigating whether *groups* show general differences in categorization performances, data were averaged separately by categories *membership* status, *round* and subject ID. As the interpretation of main effects and interactions with DLs is only meaningful for category members in this task, corresponding exploratory analyses were based exclusively on performances for category members. Distortion‐level analyses on data from the transfer phase were restricted to novel category members to circumvent biasing due to an imbalanced distribution of items shown during training (‘training items’) and novel transfer items (‘novel items’) across distortion levels (see Figure 1A). Likewise, exploratory analyses of potential differences in categorization performances between AQ groups during the transfer phase for items shown during the training phase versus novel transfer items were limited to DL5, DL6 and DL7 items. This restriction was due to the fact that the prototype and DL4 items were only presented during the transfer phase and thus not suitable for comparison with items shown during the training phase. To simplify the regression model investigating differential contributions of AQ subscale scores, data were additionally aggregated across rounds. Formulas used in linear and linear mixed models are detailed in Supporting Information S1: Sections 2 and 3.

Sum contrasts were used for unordered factors, and polynomial contrasts were used for ordered factors. All continuous variables were *z*‐standardized before entering into the models.

## 3 Results

### 3.1 AQ Group Characteristics

**FIGURE 2:**
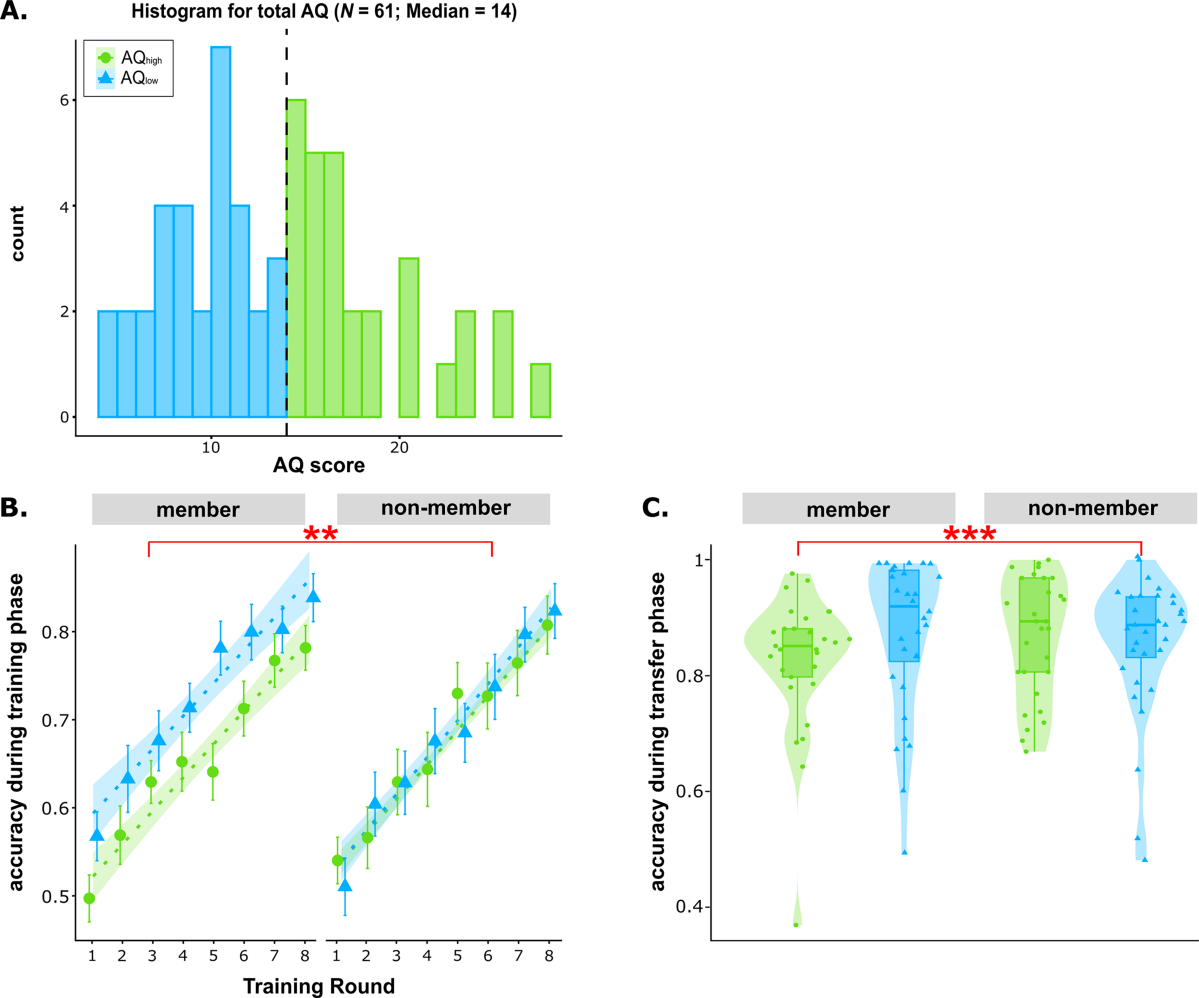
Behavioural performances during the training phase and the transfer phase. Significant main effects of group and interactions with group are highlighted with red asterisks (**p < 0.01 and ***p < 0.001). (A) Distribution of sum scores in the AQ questionnaire and visual illustration of the median split used to group individuals into low (AQ_low_) and high (AQ_high_) AQ groups. (B) Accuracy rates of categorization of members and non‐members for AQ_high_ (green circles) and AQ_low_ (blue triangles) during training. Green and blue shaded areas represent 95% confidence intervals, and error bars represent standard errors of the means. AQ_high_ showed more difficulties endorsing category members than AQ_low_, but categorization of non‐members was almost identical. (C) Accuracy rates of categorization of members and non‐members for AQ_high_ and AQ_low_ during the transfer phase. The upper and lower hinges of the boxplots represent the 25th and 75th percentiles, whiskers span 1.5 times interquartile ranges, and horizontal lines depict means. AQ_high_ showed worse performance for category members compared to AQ_low_, but not non‐members.

AQ values ranged between 4 and 28 (median = 14, *M* = 14.08, *SD* = 5.54), seemingly reflecting other subclinical samples. One systematic review of 72 studies (*N* = 6934) reported a mean of 16.94 (range: 11.6–20.0), whereas the original AQ validation article reported an average range from 11 to 20, depending on gender and occupation (Baron‐Cohen et al. 2001; Ruzich et al. 2015). To subdivide the sample into AQ groups, 32 individuals showing AQ scores smaller or equal to the median AQ were assigned to the AQ_low_ group, and 29 individuals with AQ scores above the median were assigned to the AQ_high_ group (Figure 2A). The relative proportions of female‐to‐male participants (*χ*(1) = 2.01, *p* = 0.157) or of right‐ to left‐handed participants (*χ*(1) < 0, *p* > 0.999) did not differ significantly between AQ groups. In addition, AQ groups did not differ significantly with respect to age, fluid intelligence, working memory performance, depressive symptoms, or anxiety and somatization levels (Supporting Information S2: Table 1). However, on average, the AQ_low_ group reported having completed more years of education than the AQ_high_ group (*t*(57.90) = 2.53, *p* = 0.014, 95% CI [0.27, 2.29]). Illustrating that AQ, EQ and SQ questionnaires capture partly overlapping concepts, EQ values were lower and SQ values were higher in the AQ_high_ group compared to the AQ_low_ group (Supporting Information S2: Table 1).

### 3.2 Measures of Universal Category Learning Processes

#### 3.2.1 Accuracy Rates During the Training Phase

In both groups, performances increased significantly in the course of the first eight training rounds (*t*(885) = 18.52, *p* < 0.001, 95% CI [1.14, 1.40]). However, there was a significant interaction between membership and group (*t*(885) = −2.84, *p* = 0.004, 95% CI [−0.12, −0.02]), indicating that the AQ_high_ group exhibited more difficulties correctly endorsing category members than the AQ_low_ group, whereas performances for non‐members were almost identical (Figure 2B). Accuracy for category members was negatively related to distortion levels (*t*(1357) = −3.01, *p* = 0.002, 95% CI [−0.19, −0.04]) but did not differ between AQ groups (all *p*s > 0.2).

There was no significant difference between groups in reaching the performance criterion of 0.75 accuracy when considering the full training period (i.e., up to 14 training rounds; *t*(59) = 0.78, *p* = 0.439, 95% CI [−0.16, 0.36]). Furthermore, accuracy rates in the last training round (i.e., the 8th round for the quickest learners and the 14th round for the slowest learners) did not differ between groups (main effect: *t*(59) = −0.78, *p* = 0.436, 95% CI [−0.26, 0.11]; group × membership interaction: *t*(59) = −0.80, *p* = 0.425, 95% CI [−0.27, 0.11]).

Subscale analyses were preceded by the inspection of variance inflation factors (VIFs), which verified that the interpretation of the results was not biased by the presence of high multicollinearity among predictors (VIFs < 1.5). Analyses revealed that high scores on the ‘attention to detail’ subscale of the AQ were linked to worse accuracy rates independent of category membership (*t*(55) = −2.04, *p* = 0.047, 95% CI [−0.31, −0.01]; Supporting Information S2: Figure 1A). Moreover, an interaction effect between the ‘social skills’ subscale of the AQ with membership suggested that individuals reporting more social interaction difficulties had lower accuracy rates for category members but higher accuracy rates for non‐members (*t*(55) = −2.12, *p* = 0.039, 95% CI [−0.23, −0.01], Supporting Information S2: Figure 1B).

#### 3.2.2 Accuracy Rates During the Transfer Phase

Like analyses on the first eight training rounds, analyses on data from the transfer phase yielded a group × membership interaction. That is, individuals from the AQ_high_ group exhibited worse performance for category members but not non‐members compared to the AQ_low_ group in the transfer phase (*t*(882.95) = −3.94, *p* < 0.001, 95% CI [−0.156, −0.052]; Figure 2C). As accuracy rates did not exhibit significant changes across rounds of the transfer phase (*t*(883.16) = 1.59, *p* = 0.113, 95% CI [−0.028, 0.016]), data were aggregated over rounds for all further analyses on transfer phase data. Accuracy for category members was negatively related to distortion levels (*t*(242) = −6.61, *p* < 0.001, 95% CI [−0.274, −0.149]), but did not differ between groups (*t*(242) = −0.32, *p* = 0.751, 95% CI [−0.072, 0.052]). A regression model examining the effects of ‘item novelty’ (i.e., training item vs. novel item) for DL5, DL6 and DL7 category members and non‐members showed no interactions between item novelty and group (all *p*s > 0.53).

Turning to AQ subscale analyses on transfer phase data, the inspection of VIFs verified that the interpretation of the results was not biased by the presence of high multicollinearity among predictors (all VIFs < 1.5). Similar to the training phase, scores of the ‘social skills’ subscale of the AQ interacted significantly with category membership (*t*(110) = −2.23, *p* = 0.028, 95% CI [−0.34, −0.02]). Again, individuals reporting more social interaction difficulties tended to have lower accuracy rates for category members but higher accuracy rates for non‐members (Supporting Information S2: Figure 2A). In contrast, the relationship between accuracy and scores from the ‘attention to detail’ subscale of the AQ no longer approached significance (*t*(110) = −1.38, *p* = 0.169, 95% CI [−0.26, 0.05]). Interaction terms between membership and ‘attention to detail’ scores (*t*(110) = −1.88, *p* = 0.063, 95% CI [−0.09, 0.24]) as well as scores from the ‘imagination’ subscale of the AQ (*t*(110) = −1.75, *p* = 0.084, 95% CI [−0.28, 0.02]) missed the significance threshold (Supporting Information S2: Figure 2B,C).

#### 3.2.3 Neural Correlates During the Training Phase

##### 3.2.3.1 Visual Processing and Decision‐Making

**FIGURE 3:**
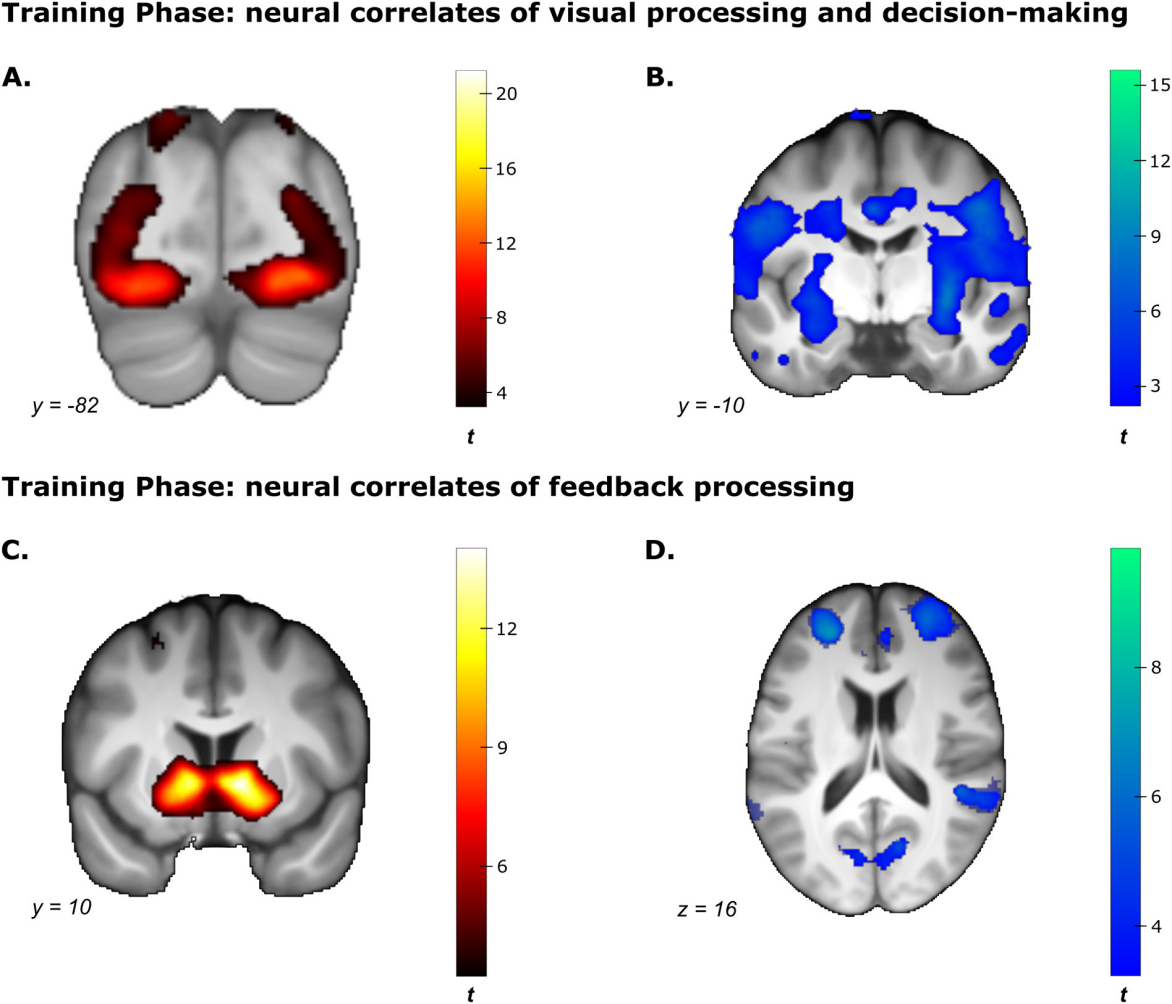
Main effects of neural correlates during the training phase during visual processing, decision‐making and feedback processing. Effects are collapsed across categories membership, training round and group. Statistical *t*‐maps are thresholded at *p*_unc_ < 0.001 for visualization. (A) Statistical *t*‐maps for the contrast of stimulus presentation against fixation cross presentation yielding a broad network of brain regions that usually handle visual processing, such as the fusiform gyrus (FUS). (B) Statistical *t*‐maps for the negative contrast of stimulus presentation against the presentation of fixation crosses showing effects, for instance, in the posterior cingulate cortex (PCC) and the insula. (C) Statistical *t*‐maps for the contrast of positive against negative feedback showing strong striatal activation presentation. (D) Statistical *t*‐maps for the contrast of negative against positive feedback, for example, yielding effects in the prefrontal cortex.

Relative to the baseline (i.e., fixation crosses), presenting stimuli during training was linked to a broad network of brain regions that usually handle visual processing, such as the medioventral and rostroventral FUS (see Figure 3A and Supporting Information S2: Table 2). Additionally, these regions included those that engage in visuo‐spatial attention, such as the lateral superior parietal cortex (e.g., *Z* = 10.75, *p*_unc_ < 0.001, *p*_cor_ < 0.001, *x* = −34, *y* = −44, *z* = 46), as well as regions dealing with perceptual decision‐making, such as the frontal cortices and the anterior insula (see Supporting Information S2: Table 2). The PCC was significantly less activated during stimulus presentation compared to baseline (see Figure 3B and Supporting Information S2: Table 2). Contrasting non‐members against category members revealed stronger responses in the caudal cuneus and lingual gyrus. Whole‐brain analyses did not reveal significant differences between groups (largest effect: *Z* = 4.58, *p*_unc_ < 0.001, *p*_cor_ = 0.159 in the middle temporal cortex).

**FIGURE 4:**
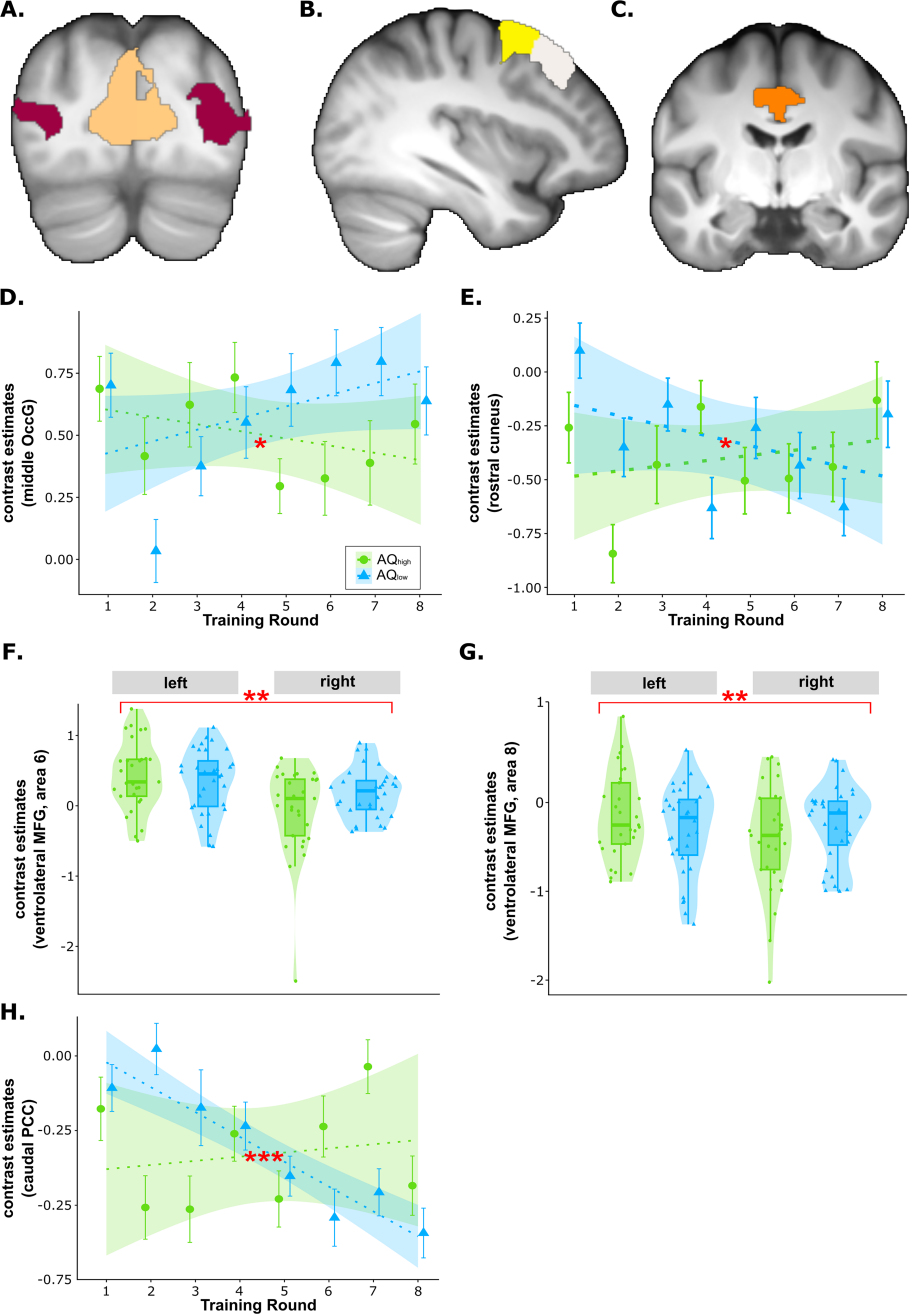
Anatomical masks and results of ROI‐based analyses during the training phase for visual processing and decision‐making. Significant interactions between group and training round or hemisphere are highlighted with red asterisks (*p < 0.05, **p < 0.01 and ***p < 0.001). (A) Anatomical mask of regions involved in visual processing: the rostral cuneus (peach colour) and the middle occipital gyrus (OccG; cherry colour). (B) Anatomical mask of Area 6 of the ventrolateral midfrontal gyrus (MFG; yellow) and Area 6 of the ventrolateral MFG (white), typically involved in decision‐making. (C) Anatomical mask of the caudal posterior cingulate cortex (PCC; orange colour), typically involved in decision‐making. (D) Contrast estimates from the middle OccG for AQ_high_ and AQ_low_ across training rounds, indicating a decrease over training in AQ_high_ but an increase in AQ_low_. (E) Contrast estimates from the rostral cuneus for AQ_high_ and AQ_low_ across training rounds, indicating an increase over training in AQ_high_ but a decrease in AQ_low_. (F) Contrast estimates in the ventrolateral MFG (Area 6) for AQ_high_ and AQ_low_ across training rounds, indicating lateralization differences between AQ_high_ and AQ_high_. (G) Contrast estimates from the ventrolateral midfrontal gyrus (MFG; Area 8) for AQ_high_ and AQ_low_ across training rounds, revealing lateralization differences between AQ_high_ and AQ_high_. (H) Contrast estimates from the caudal PCC for AQ_high_ and AQ_low_ across training rounds, revealing an increase over training in AQ_high_ but a decrease in the AQ_low_ group.

Analyses based on anatomical ROIs typically associated with visual processing (e.g., Figure 4A) indicated a group × round interaction for relative activation in the middle occipital gyrus (OccG; *t*(1829) = −3.10, *p*_unc_ = 0.002, *p*_cor_ = 0.031, 95% CI [−0.232, −0.052]) and in the rostral cuneus (*t*(1829) = 3.32, *p*_unc_ < 0.001, *p*_cor_ = 0.014, 95% CI [0.066, 0.257]). Activation of the middle OccG slightly decreased over training in the AQ_high_ group but increased in the AQ_low_ group (Figure 4D). Rostral cuneus activation slightly increased in the AQ_high_ group during training but decreased in the AQ_low_ group (Figure 4E). Analyses based on anatomical ROIs typically associated with decision‐making (e.g., Figure 4B,C) revealed a group × hemisphere interaction in Area 6 of the ventrolateral MFG (*t*(1829) = 3.81, *p*_unc_ < 0.001, *p*_cor_ = 0.002, 95% CI [0.121, 0.340]) (Figure 4F) and in Area 8 of the ventrolateral midfrontal gyrus (MFG; *t*(1829) = 3.41, *p*_unc_ < 0.001, *p*_cor_ = 0.009, 95% CI [0.029, 0.106]) (Figure 4G), suggesting lateralization differences between groups. Moreover, although caudal PCC activation remained relatively stable in the course of training in the AQ_high_ group, it decreased in the AQ_low_ group (*t*(1829) = 5.48, *p*_unc_ < 0.001, *p*_cor_ < 0.001, 95% CI [0.192, 0.405]; Figure 4H). To investigate whether group differences in the explicit baseline (i.e., fixation cross onsets) could have mediated the above‐mentioned effects, activity related to fixation cross onsets was contrasted against the explicit baseline as a further control. Analyses did not hint towards significant group differences in baseline activity (all *p*_cor_ > 0.255).

##### 3.2.3.2 Feedback Processing

The presentation of positive relative to negative feedback evoked stronger activity in the VS, the dorsal striatum, the ventral PCC and some frontal gyri (Figure 3C and Supporting Information S2: Table 2). Negative feedback was associated with stronger activity in the SFG, MFG and IFG frontal regions (Figure 3D and Supporting Information S2: Table 2). Two‐sample *t*‐tests on the whole scan volume did not show significant differences between groups when comparing positive against negative feedback (largest effect: *Z* = 4.02, *p*_unc_ < 0.001, *p*_cor_ = 0.737 in white matter) or vice versa (largest effect: *Z* = 4.35, *p*_unc_ < 0.001, *p*_cor_ = 0.329 in white matter).

**FIGURE 5:**
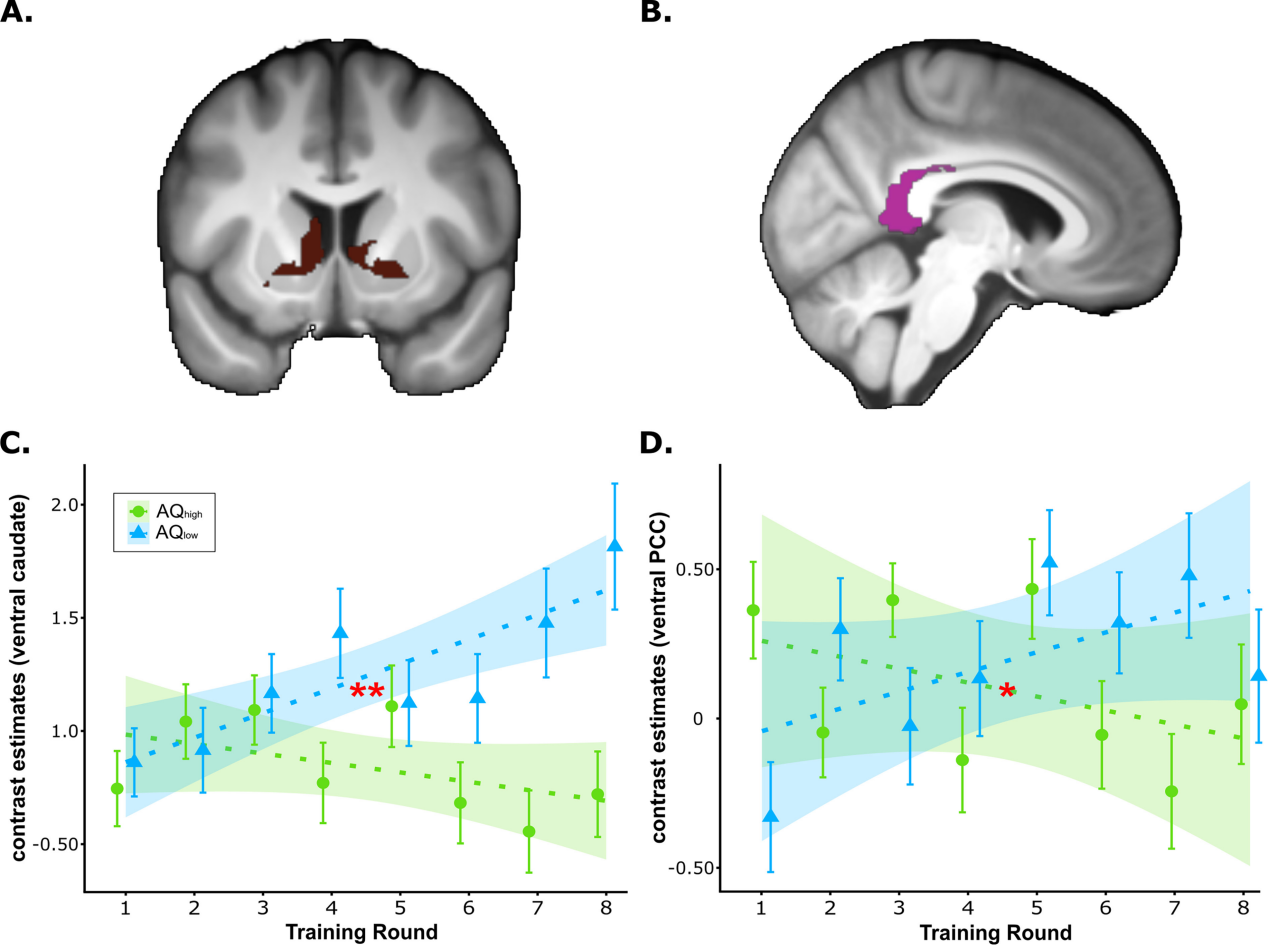
Anatomical masks and results of ROI analyses during the training phase during feedback processing. Significant interactions between group and training round are highlighted with red asterisks (*p < 0.05 and **p < 0.01). (A) Anatomical mask of the ventral caudate (brown). (B) Anatomical mask of the ventral posterior cingulate cortex (PCC; pink). (C) Contrast estimates from the ventral caudate for AQ_high_ and AQ_low_ across training rounds, indicating a decrease over training in AQ_high_ but an increase in AQ_low_. (D) Contrast estimates from the ventral PCC for AQ_high_ and AQ_low_ across training rounds, indicating a decrease over training in AQ_high_ but an increase in AQ_low_.

Analyses based on anatomical ROIs typically associated with feedback processing (e.g., Figure 5A,B) revealed a significant group × round interaction in the ventral caudate within the VS (*t*(1698.00) = −3.99, *p*_unc_ < 0.001, *p*_cor_ = 0.002, 95% CI [−0.360, −0.123]; Figure 5C) and in the ventral PCC (*t*(1709.13) = −3.20, *p*_unc_ = 0.001, *p*_cor_ = 0.035, 95% CI [−0.325, −0.078]; Figure 5D), with a relative activity decrease in the AQ_high_ group and an increase in the AQ_low_ group in the course of training.

#### 3.2.4 Neural Correlates of Visual Processing and Decision‐Making During the Transfer Phase

The presentation of to‐be‐categorized stimuli was related to regions typically associated with higher visual processing, such as the medioventral and rostroventral FUS, as well as regions typically involved in decision‐making like frontal regions, the anterior insula and cingulate gyri (Supporting Information S2: Table 3). Non‐members were associated with stronger activation in the occipital polar cortex and the caudal lingual gyrus. Neither whole‐brain analyses (largest effect: *Z* = 4.26, *p*_unc_ < 0.001, *p*_cor_ = 0.398 in the ITG) nor analyses on estimates from ROIs typically associated with visual processing revealed significant group differences (largest effect: *t*(177) = −2.93, *p*_unc_ = 0.004, *p*_cor_ = 0.056 in the ITG). Analyses based on ROIs typically associated with decision‐making revealed a group × hemisphere interaction in the ventrolateral MFG (*t*(177) = 4.72, *p*_unc_ < 0.001, *p*_cor_ < 0.001, 95% CI [0.104, 0.253]), with a weaker right lateralized activation in the AQ_high_ group compared to the AQ_low_ group. Group × hemisphere interactions were evident in the opercular IFG (*t*(177.00) = 3.01, *p*_unc_ = 0.003, *p*_cor_ = 0.042, 95% CI [0.040, 0.188]) and the anterior insula (*t*(177) = 4.07, *p*_unc_ < 0.001, *p*_cor_ = 0.001, 95% CI [0.073, 0.209]), both with stronger activations in the AQ_high_ compared to the AQ_low_ in the left hemisphere.

Control analyses (Supporting Information S2: Section 7) showed that group differences in baseline activity contributed significantly to all group × hemisphere interactions for which group effects were found, namely, in the ventrolateral MFG (*p*_cor_ = 0.027), the opercular IFG (*p*_cor_ = 0.025) and the anterior insula (*p*_cor_ = 0.015). Given that the integrity of the decision‐making group effects is questioned by these baseline effects, these particular group effects are not discussed further, in order to avoid over‐interpretation.

### 3.3 Measures of Category Learning Strategies

#### 3.3.1 Model Fits and Parameter Estimates for Behavioural Training Phase Data

**FIGURE 6:**
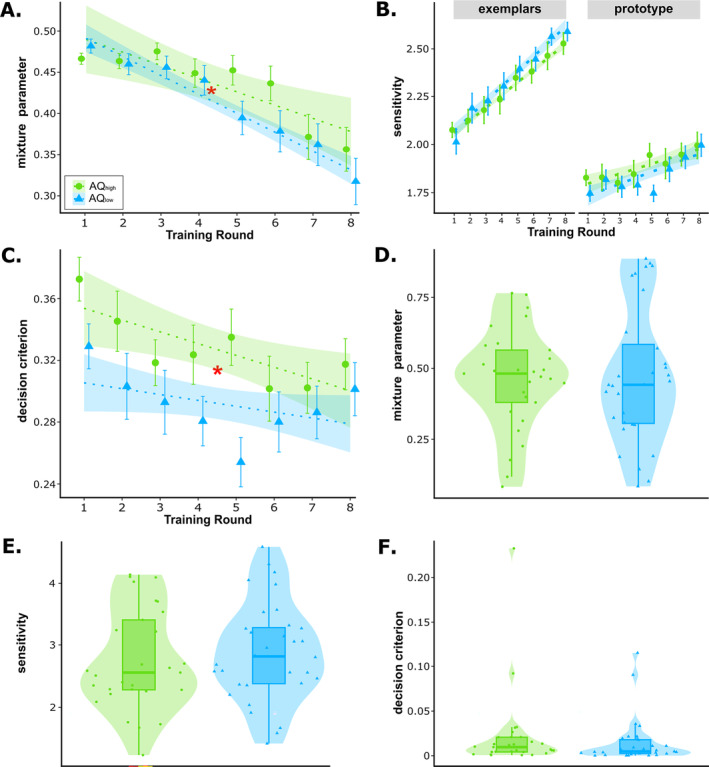
Parameter estimates from mixture models. Significant main effects of group and interactions with group are highlighted with red asterisks (*p < 0.05). (A) Estimated mixture parameter for the training phase. AQ_high_ (green circles) and AQ_low_ (blue triangles) during training. Green and blue shaded areas represent 95% confidence intervals, and error bars represent standard errors of the means. Results show a less pronounced, that is, slower, development away from the prototype strategy in the AQ_high_ group. (B) Estimated sensitivity during the training phase. (C) Estimated decision criterion during the training phase, revealing that AQ_high_ was on average stricter than AQ_low_. (D) Estimated mixture parameter during the transfer phase. The upper and lower hinges of the boxplots represent the 25th and 75th percentiles, the whiskers span 1.5 times the interquartile ranges, and horizontal lines depict means. (E) Estimated sensitivity during the transfer phase. (F) Estimated decision criterion during the transfer phase.

The MIX‐2c‐*γ* model showed the best fit to training data relative to the guessing model (see Supporting Information S2: Table 4). At the very beginning of the training phase, individuals made on average almost equal use of both the exemplar and prototype strategies (training Round 1: *M*_*β*_ = 0.474, *SD*_*β*_ = 0.043). As training proceeded, individuals from both groups successively relied less on the prototype strategy (*t*(413) = −11.05, *p* < 0.001, 95% CI [−1.18, −0.83]). However, analyses revealed a significant group × round interaction (*t*(413) = 2.43, *p* = 0.015, 95% CI [0.004, 0.402], with a less pronounced development away from the prototype strategy in the AQ_high_ group (Figure 6A). Sensitivity to the prototype and exemplars both increased in the course of training (prototype: *t*(413) = 5.25, *p* < 0.001, 95% CI [0.360, 0.782]; exemplar: *t*(413) = 16.16, *p* < 0.001, 95% CI [1.06, 1.35]), but neither differed between groups (all *p*s > 0.382; Figure 6B). Exploratory analyses on the decision criterion for endorsing an item as a category member showed that decisions became more liberal in the course of training (*t*(413) = −3.55, *p*_cor_ < 0.001, 95% CI [−0.625, −0.180]) and were on average stricter in the AQ_high_ group (*t*(413) = 2.40, *p*_cor_ = 0.038, 95% CI [0.033, 0.326]; Figure 6C). In addition, the response scaling parameter indicated more deterministic choices in the course of training (*t*(413) = 21.91, *p*_cor_ < 0.001, 95% CI [1.41, 1.69]), but no significant group differences (all *p*_cor_s > 0.154).

#### 3.3.2 Model Fits and Parameter Estimates for Behavioural Transfer Phase Data

The MIX model showed the best fit to data from the transfer phase relative to a guessing model (see Supporting Information S2: Table 5). Groups did not differ significantly in the tendency to prefer the prototype over the exemplar strategy (or vice versa) between AQ groups (*t*(59) = 0.38, *p* = 0.704, 95% CI [−0.228, 0.338]; Figure 6D). Likewise, there were no group differences in sensitivity to the prototype or exemplars, which is not separable in this model (*t*(59) = −0.62, *p* = 0.539, 95% CI [−0.378, 0.197]; Figure 6E). Exploratory analyses on the response criterion also did not yield a significant group difference (*t*(59) = 0.953, *p* = 0.344, 95% CI [−0.037, 0.107]; Figure 6F).

#### 3.3.3 Combined Neural Prototype/Exemplar Representations During the Training Phase

**FIGURE 7:**
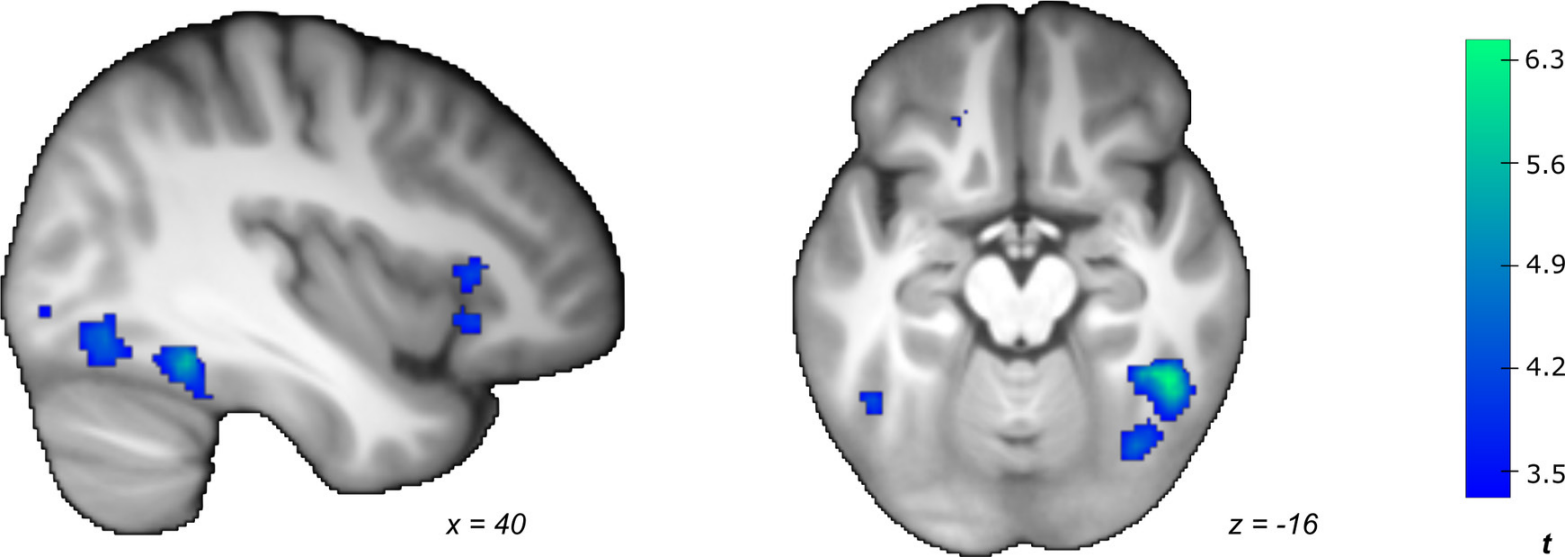
Negative neural correlates for combined prototype–exemplar representation during the training phase, mainly located in the medioventral and lateroventral fusiform gyrus (FUS). Effects are collapsed across categories membership, training round and group. Statistical *t‐*maps are thresholded at *p*_unc_ < 0.001 for visualization.

Similarity to the prototype/exemplars was negatively associated with activity in the medioventral and rostroventral FUS (Figure 7), the occipital polar cortex and the caudal cuneus (Supporting Information S2: Table 2). No region showed a positive relationship with similarity to prototype/exemplars during training. Two‐sample *t*‐tests on the whole scan volume did not show significant group differences (largest effect: *Z* = 3.88, *p*_unc_ < 0.001, *p*_cor_ = 0.875 in the caudodorsal cingulate gyrus).

**FIGURE 8:**
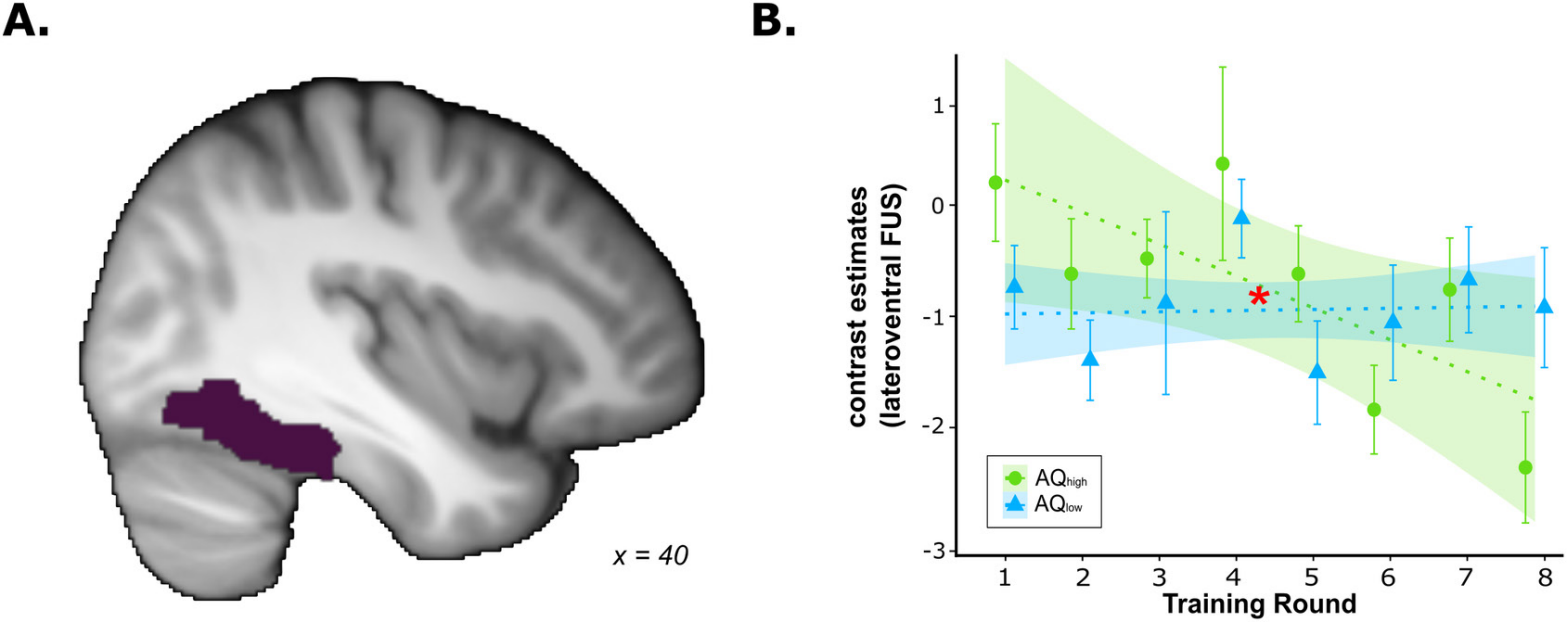
Anatomical mask and ROI analysis of combined exemplar/prototype representations. (A) Anatomical mask of the lateroventral fusiform gyrus (FUS; plum colour), which is typically involved in prototype representation. (B) Contrast estimates from the lateroventral FUS for AQ_high_ and AQ_low_ during the training phase. The significant interaction between group and training round is highlighted with a red asterisk (*p < 0.05). The figure illustrates that although recruitment of this region in AQl_ow_ remained relatively stable, it became more pronounced in the AQ_high_ group.

Analyses based on anatomical ROIs typically associated with univariate prototype/exemplar representations (e.g., Figure 8A) indicated that the training‐related change in the association between similarity to prototype/exemplars and neural activity differed between groups in the lateroventral FUS (*t*(881.45) = −3.10, *p*_unc_ = 0.002, *p*_cor_ = 0.016, 95% CI [−0.354, −0.080]; Figure 8B). Although negative prototype/exemplar correlates in the AQ_high_ group became stronger during the course of training, prototype/exemplar correlates in the AQ_low_ group remained relatively stable.

Exploratory ROI‐based analyses within IPS subregions previously associated with multivariate prototype/exemplar representations yielded an interaction between group and round in the lateral superior parietal lobe (SPL), which was not significant following multiple‐comparison corrections (*t*(881.28) = −2.87, *p*_unc_ = 0.004, *p*_cor_ = 0.055, 95% CI [−0.372, −0.070]; Supporting Information S2: Figure 3). Although the AQ_high_ group showed increasingly negative prototype/exemplar representations across training rounds, negative prototype/exemplar representations in the AQ_low_ group became less pronounced over training.

#### 3.3.4 Neural Prototype and Exemplar Representations During the Transfer Phase

No regions showed positive relationships with similarity to the prototype. Similarity to the prototype correlated negatively with activity in the lateral inferior OccG and the lateroventral FUS (Supporting Information S2: Table 3). Similarity to exemplars correlated positively with activity in the occipital polar cortex, the posterior inferior OccG, and the medioventral and rostroventral FUS. Similarity to exemplars was negatively correlated with activity in the medioventral OCC. Neither two‐sample *t*‐tests on the whole scan volume (largest effect: *Z* = 4.30, *p*_unc_ < 0.001, *p*_cor_ = 0.360 in the paracentral lobule) nor analyses within anatomical ROIs typically associated with univariate prototype/exemplar representations (all *p*_cor_ > 0.160) showed significant group differences.

In exploratory ROI‐based analyses within IPS subregions previously associated with multivariate prototype/exemplar representations, a significant interaction was found between group and round for contrast estimates from a caudal SPL mask. This indicated that only the AQ_high_ group exhibited a pronounced negative correlation between prototype similarity and activity in the left hemisphere (*t*(59) = −3.50, *p*_unc_ < 0.001, *p*_cor_ = 0.001, 95% CI [−0.271, −0.076]; Supporting Information S2: Figure 3).

## 4 Discussion

A subclinical sample of individuals high in autistic traits was significantly worse at endorsing category members than individuals low in autistic traits, expanding previous research on categorization difficulties in autistic samples. Analyses revealed several factors that could explain such categorization difficulties. Groups differed in neural correlates related to visual processing in occipital regions, decision‐making in midfrontal regions and the caudal PCC, and feedback processing in the ventral PCC and the ventral caudate in the VS during the training phase, suggesting a link between autistic traits and alterations in universal subprocesses of categorization. Model‐based analyses did not indicate an attenuated autistic trait‐related usage of the prototype strategy, contradicting our hypothesis that a prototype abstraction deficit underlies categorization difficulties. Instead, model‐based analyses showed that individuals high in autistic traits appeared to have stricter decision policies over the course of training. We additionally observed group differences in combined prototype/exemplar neural representations in the ventrolateral FUS during training, indicating a difference in how the brain develops category representations in higher visual regions.

In our study, neurotypical individuals high in autistic traits showed worse categorization performances, as predicted by Hypothesis 1. This connects well to previous research showing categorization difficulties for abstract (Church et al. 2010; Gastgeb et al. 2012) and facial stimuli in autistic individuals (Strauss et al. 2012). The successful reproduction of categorization difficulties in a well‐controlled, neurotypical sample confirms that categorization difficulties are not an artefact of psychiatric comorbidities prevalent in autistic samples and are in fact related to autistic traits on the BAP, not just on a clinical level. In addition, our approach of excluding individuals who performed at chance level argues against the suspicion that autism‐related differences in perceptual categorization are merely the result of a higher number of at‐chance performances among autistic individuals raised by a reanalysis of a dataset in autistic children (Church et al. 2010; Voorspoels et al. 2018). Nevertheless, the translation of results from a subclinical sample to a clinical population should be considered cautiously. Behavioural autistic traits, as measured in questionnaires, do not represent the complex neurotype of autism itself (Sasson and Bottema‐Beutel 2022). That said, studies replicating findings from ASC in the BAP can be informative for understanding the mechanisms and interactions of co‐occurring traits for both autists and the general population (Landry and Chouinard 2016). Alternatively, studies in neurotypical subjects that fail to replicate findings in ASC allow us to recognize attributes unique to ASC, perhaps due to its neurodevelopmental differences (Landry and Chouinard 2016).

The current study found that group differences in endorsement probabilities were confined to category members, indicating that different mechanisms may underlie the categorization of members and non‐members. This moderation of group differences by category membership could be related to different stimulus characteristics for members compared to non‐members. As category membership is defined by some degree of overall similarity among stimuli, non‐members are randomly generated and therefore necessarily much more distinct from each other. Consequently, the categorization of category members depends much more on comparing stimuli to a mental representation of the category, whereas for non‐members, distinctiveness or saliency compared to all other items may serve as an additional discriminative cue. Speaking in Bayesian and predictive coding terms, the appropriate shaping of prior beliefs (i.e., the category's representation) and the down‐weighting of prediction errors (i.e., category‐irrelevant deviations of the category's representation) are more critical to correctly identify members than to reject non‐members. Difficulties with social skills were positively correlated with endorsing category members and negatively correlated with endorsing non‐members, supporting a link towards an autism‐specific cognitive processing style and social difficulties (Sinha et al. 2014). Contrary to social difficulties, attention to detail scores were negatively related to the categorization of both members and non‐members. One could assume that individuals with a high focus to details not only have difficulties ignoring category‐irrelevant deviations from the category's mental representation but also have more difficulties processing stimuli in relation to each other, so that individuals have more difficulties processing both similarity and distinctiveness. This aligns well with the WCC theory that autistic individuals have difficulties integrating information into a meaningful whole or the *gist* of a set, but that local processing remains intact (Frith and Happé 1994; Happé and Booth 2008; Happe and Frith 2006; Koldewyn et al. 2013). This is also in agreement with the alternative EPF theory (Burack et al. 2001; Mottron et al. 2006; Van Eylen et al. 2018) that individuals with ASD rather have a bias towards local processing partially because of a superiority in this system and not necessarily a deficit in global processing.

The replication of previous clinical results in a neurotypical sample, as well as previous theories of ASC, holds implications for all individuals showing high autistic traits, that is, those in the BAP, as well as with ASC. In accordance with Hypothesis 2, higher ‘attention to detail’ scores were linked to worse categorization performance. This was also true for difficulties with social interaction. Therefore, it appears that having higher autistic traits may translate to difficulties in local–global processing during everyday life, including in social situations. Literature suggests that individuals specifically scan social encounters for similarities or familiar categories (e.g., tonal inflections and facial expressions), which then guide an appropriate response selection based on previous social interactions (Haker, Schneebeli, and Stephan 2016; Liberman, Woodward, and Kinzler 2017; Sinha et al. 2014). Previous theories suggest that individuals high in autistic traits might have difficulties separating context‐relevant information from noise, leading to greater prediction error, that is, inappropriate responses, during these interactions (Van de Cruys et al. 2014; Van de Cruys, Perrykkad, and Hohwy 2019). To summarize, the behavioural data suggest separate processing mechanisms for members and non‐members, given that individuals high in autistic traits only showed perceptual categorization difficulties of members. Examining the imaging data could illuminate some of the potential mechanisms behind these differences.

Analyses of event‐related fMRI data suggested a link between autistic traits and all universal subprocesses of category learning specifically during training. In regions of visual processing, AQ_high_ recruited the middle OccG progressively less and the cuneus within the posterior OCC progressively more during training, whereas AQ_low_ individuals showed the opposite. Previous research found enhanced activity in both the cuneus and other primary visual areas in autistic compared to neurotypical participants (Fan et al. 2021; Kovarski et al. 2019; Ring et al. 1999), and research based on the predictive coding framework has shown that these areas are sensitive to predictability (Alink et al. 2010). Further evidence suggests that autistic individuals rely more on lower level visual information for processing association strength and relevance to a category, supported by findings that autistic individuals show enhanced activation of visuo‐perceptual regions during perceptual processing compared to neurotypicals (Mottron et al. 2006; Wong, Gau, and Chou 2019). Higher level visual areas such as the middle OccG, on which neurotypical individuals with low autistic traits are thought to be more reliant, are responsible for the holistic processing of stimuli (Todorova, Pollick, and Muckli 2021). It is thus plausible that individuals high in autistic traits have reduced abilities to adapt to task requirements in terms of switching from lower to higher level visual processing so that it is more difficult to process stimuli in a holistic way and down‐weight prediction errors caused by category‐irrelevant details. Alternatively, the EPF theory posits that activation of higher level visual areas may be ‘optional’ (as opposed to impaired) in ASC during categorization, when lower level areas will suffice (Mottron et al. 2006; Van Eylen et al. 2018).

Turning to decision‐making, the AQ_low_ group showed a pronounced decrease in the recruitment of the caudal PCC, with caudal PCC involvement in the AQ_high_ group staying at a relatively constant low level. Atypicalities in the PCC have previously been described in autistic individuals (Leung and Lau 2020; Leech and Sharp 2014). The PCC is part of a choice evaluation network in risky decision‐making (Engelmann and Tamir 2009; Lim, O'Doherty, and Rangel 2011), coding for perceived risk and mediating updating of choice alternative values (Heilbronner, Hayden, and Platt 2011; Kobayashi and Hsu 2017). The low involvement of the caudal PCC in the AQ_high_ group could thus cause categorization difficulties through inappropriate risk perception or value updating, whereas the progressing reduction in caudal PCC activity in the AQ_low_ group likely mirrors reduced subjective risk appraisal and reduced necessity to update values when performances improve. In addition, we found different lateralization patterns in the ventrolateral MFG between groups. Atypicalities in lateralization in frontal and other regions have been already reported in ASC and have been proposed to be connected to altered communication between the two hemispheres (Leisman, Melillo, and Melillo 2023; Persichetti et al. 2022).

Regarding feedback processing, the AQ_high_ group showed decreases in neural responses to positive relative to negative feedback in the ventral PCC and the ventral caudate during training, whereas the AQ_low_ group exhibited increases in these regions. Given that previous research shows a role of the ventral PCC in memory retrieval (Foster et al. 2023; Leech and Sharp 2014) and that this region showed stronger activation for positive relative to negative feedback in our study, it is plausible that the recruitment of the ventral PCC reflects a greater attempt to memorize specific items as members or non‐members after positive relative to negative feedback (Bzdok et al. 2015). One could theorize that individuals low in autistic traits focused more on memorizing those patterns for which they received positive feedback, whereas individuals high in autistic traits took the opposite approach.

Concerning the ventral caudate, it is known that this region is involved in the processing of reward, motivation and task success, going well with our finding of stronger activation for positive relative to negative feedback. Therefore, stronger activation of the ventral caudate during the training phase could reflect increases in reward‐related learning, overweighting the informative value of positive versus negative feedback, or positive feedback being experienced as progressively more rewarding and motivating, as well as aiding task success (Haruno et al. 2004; Tricomi and Fiez 2008). Both groups showed nearly identical, relatively low VS activation during the beginning of training, though only the AQ_low_ group increased activation over time. This aligns with previous findings that individuals with ASC underreact to typical stimuli and therefore benefit less from feedback (Kohls et al. 2013).

In summary, and in concurrence with Hypothesis 3, we found evidence for a link between autistic traits and the adaptation of neural processes supporting visual processing, decision‐making and feedback processing during category learning.

Beyond looking at the different subprocesses of category learning through event‐related fMRI, model‐based analyses were utilized to generate further insight into what underlies categorization difficulties. Contrary to observations of performances in autistic children during the transfer phase of a similar category task (Church et al. 2010) and to our Hypotheses 4 and 5, we not only failed to find support that individuals high in autistic traits exhibit difficulties in prototype abstraction but even found a higher reliance on prototype strategy in the course of training. In opposition to our hypotheses and the findings from Church et al. (2010), an aversion to the prototype strategy therefore cannot be the reason for categorization difficulties observed in this sample. It is however worth noting that, unlike Church et al., we chose a subclinical sample and also excluded individuals who did not perform above chance, given that a reanalysis separating at‐ and above‐chance performances could not support significantly lowered sensitivities to the prototype in autism (Voorspoels et al. 2018). Nevertheless, the finding of increased reliance on the prototype strategy in individuals high in autistic traits warrants further exploration.

Although strong relationships between similarity to the prototype and accuracy, as well as between prototype similarity and activity in certain brain areas like the FUS, are well evidenced (Blank and Bayer 2022; Lech, Güntürkün, and Suchan 2016), the exact cognitive processes measured by the prototype model are far from being completely understood. Critically, it is not known how well a simple prototype model, as in our study, differentiates a pure implicit prototype process from the contribution of other more explicit category learning strategies such as rule‐based learning. In our task, a subject may have used an additional explicit verbalizable rule such as ‘any item that looks like a bird belongs to Category A’. As individuals with autism are known to sometimes follow such rules more rigidly (Petrolini, Jorba, and Vicente 2023), this could explain why individuals high in autistic traits deviated even more slowly from the prototype strategy. This also goes well with our finding of stricter decision criteria in individuals high in autistic traits, fitting to observations in autistic individuals (Pirrone et al. 2017, 2020). As we explicitly instructed participants to process stimuli in a holistic way, it is well plausible that our instruction stimulated such a ‘rule‐based prototype strategy’ in high autistic trait individuals. On the neuronal level, negatively signed correlates of similarity to the prototype and/or exemplars in the FUS became stronger during training of the AQ_high_ group, whereas the correlates in the AQ_low_ group remained relatively stable. Based on the finding that activity in the FUS correlated negatively with similarity to the prototype and positively with similarity to exemplars when the two models are estimated separately (Blank and Bayer 2022), one can assume that the negative effect in the FUS could reflect neural representations of the prototype. This fits to the behavioural finding that individuals high in autistic traits averted more slowly from the prototype strategy during training than AQ_low_ individuals.

In additional exploratory ROI‐based fMRI analyses, we found that the AQ_high_ group exhibited a progressively stronger negative correlation between similarity to the prototype and activity in a subregion of the IPS. In addition, AQ_high_ showed a pronounced negative correlation, specifically within a subregion of the IPS in the left hemisphere. As the IPS showed a relationship to the distance from the decision bound in a similar category learning task (Braunlich, Liu, and Seger 2017), and univariate prototype representations in this area have not been found in this or previous studies (Blank and Bayer 2022; Braunlich, Liu, and Seger 2017), it is plausible that these observations rather mirror stricter decision policies in the AQ_high_ group than alterations in prototype representation.

In summary, our data appear to support Bayesian and predictive coding explanations of autism‐related categorization difficulties. However, a deeper understanding could be gained through the additional use of tasks and methodologies that allow to investigate Bayesian and predictive coding processes as well as rule‐based categorization.

## 5 Methodological Considerations

Some limitations of this study should be considered. Firstly, the continuous AQ values were separated according to a median split. There are potential risks with categorizing continuous variables, for example, possible loss of information concerning relative frequencies within the data and effects found within artificial thresholds (Bissonnette et al. 1990; DeCoster, Gallucci, and Iselin 2011; Iacobucci et al. 2015). However, given the complexity of our dataset, this median split allowed optimization of statistical power, increased interpretability and avoided an overly complex model (DeCoster, Gallucci, and Iselin 2011). In addition, this method has been used effectively in similar studies, helping us to position our findings within the context of existing research (Alink and Charest 2020; O'Keefe and Lindell 2013; Skewes, Jegindø, and Gebauer 2015).

There is also a risk of overgeneralizing results in subclinical samples to autistic individuals (Sasson and Bottema‐Beutel 2022). As mentioned above, a study of autistic traits is not a direct examination of ASC, though understanding how these traits interact with one's environment may still have implications for patients. Here, we show variation even in subclinical presentation, demonstrating that the interpretation of said traits also holds relevance for the BAP.

Additionally, despite convincing support that atypicalities in predictive processes could contribute to categorization difficulties, our task was not designed to explicitly examine these processes. Our task was also not suitable to investigate rule‐based learning, and subjects may have used other strategies unaccounted for here. Moreover, the timing of the task may not have been optimal for detecting subtle effects of feedback processing. This timing was deliberately chosen to avoid further extending the already lengthy task duration, especially given our intention to use a similar task to investigate ASC and gender differences in future research. Despite these limitations, the robust main effects of positive versus negative feedback suggest that the timing was adequate to capture the broader impact of feedback processing within the task. Future research could refine timing parameters to identify these nuanced effects more sensitively. Finally, group differences found for fMRI data from the transfer phase were contaminated by baseline differences, so it remains unknown to which degree autistic traits had an impact on visual processing or decision‐making in the transfer phase.

## 6 Conclusions

We found that perceptual categorization difficulties also occur in neurotypical individuals high in autistic traits. Although our data do not support the idea that a deficit in prototype abstraction accounts for categorization difficulties, autistic trait‐related group differences in neural correlates of visual processing, decision‐making and feedback processing during training suggest that atypicalities in multiple universal category learning processes could explain accuracy differences. Several results fit well to predictive coding approaches of autism, but more research is necessary as to whether atypicalities in predictive coding can explain the pattern of results completely. In addition, it remains open whether an alternative untested strategy such as inappropriate rule‐based categorization explains our finding of an even stronger reliance on the prototype strategy in the course of training and how this connects to categorization difficulties.

## Author Contributions

**Claire V. Warren:** formal analysis, writing – original draft, writing – review and editing. **Rebekka Baumert:** formal analysis, investigation, writing – review and editing. **Kira Diermann:** investigation, methodology, writing – review and editing. **Daniel Schöttle:** conceptualization, writing – review and editing. **Janine Bayer:** conceptualization, data curation, formal analysis, funding acquisition, methodology, project administration, supervision, writing – original draft, writing – review and editing.

## Conflicts of Interest

The authors declare no conflicts of interest.

### Peer Review

The peer review history for this article is available at https://www.webofscience.com/api/gateway/wos/peer‐review/10.1111/ejn.70000.

## Supporting information


**Supporting Information S1** Supplementary Methods.

**Supporting Information S2** Supplementary Results.

## Data Availability

Data will be made available upon reasonable request to the authors.
