# Supplementary material for: Linking Subclinical Autistic Traits and Perceptual Category Learning
Source: Eur J Neurosci. 2025 Feb 17;61(4):e70000. doi: 10.1111/ejn.70000 (PMC11831246; doi:10.1111/ejn.70000)
Supplement: Supplementary file 1 — Supporting Information S1 Supplementary Methods. [file EJN-61-0-s002.docx]

# Linking subclinical autistic traits and perceptual category learning

## Supplementary Methods

Claire Warren, Rebekka Baumert, Kira Diermann, Daniel Schöttle, Janine Bayer

## Regions of Interest

Supplementary Methods, Table 1. Anatomical regions of interest.

|  | **region** | **subregions** | **BNA labels** |
| --- | --- | --- | --- |
| ***visual processing*** | posterior occipital cortex | caudal lingual gyrus, rostral & caudal cuneus; occipital polar cortex | 189 - 194; 203 - 204 |
|  | lateral occipital cortex | middle & inferior occipital gyrus (OccG) | 199 - 200; 205 - 206 |
|  | fusiform gyrus (FUS) | rostroventral, medioventral & lateroventral FUS | 103 - 108 |
|  | inferior temporal gyrus (ITG) | intermediate ventral, extreme lateroventral, rostral, intermediate lateral, ventrolateral, caudolateral & caudoventral ITG | 89 - 102 |
| ***decision making*** | superior frontal gyrus (SFG) | medial SFG | 1 - 2 |
|  | middle frontal gyrus (MFG) | dorsal MFG; inferior frontal junction; area 46; ventral, ventrolateral (areas 8 & 6) & lateral MFG | 15 - 28 |
|  | inferior frontal gyrus (IFG) | opercular IFG | 37 - 38 |
|  | anterior insula |  | 167 - 168 |
|  | ventromedial putamen |  | 225 - 226 |
|  | posterior cingulate cortex (PCC) | dorsal, ventral & caudal PCC | 175 - 176; 181 - 182; 185 - 186 |
| ***feedback processing*** | superior frontal gyrus (SFG) | medial, dorsolateral (areas 8 & 6), lateral, medial (areas 6, 9 & 10) SFG | 1 - 14 |
|  | middle frontal gyrus (MFG) | dorsal MFG | 15 - 16 |
|  | inferior frontal gyrus (IFG) | dorsal IFG; inferior frontal sulcus; caudal, rostral, opercular & ventral IFG | 29 - 40 |
|  | inferior temporal gyrus (ITG) | intermediate ventral, extreme lateroventral, rostral, intermediate lateral, ventrolateral, caudolateral & caudoventral ITG | 89 - 102 |
|  | anterior cingulate cortex (ACC) | pregenual & subgenual ACC | 179 - 180; 187 - 188 |
|  | middle cingulate cortex |  | 183 - 184 |
|  | posterior cingulate cortex (PCC) | dorsal, ventral & caudal PCC | 175 - 176; 181 - 182; 185 - 186 |
|  | ventral striatum | ventral caudate; nucleus accumbens | 219 - 220; 223 - 224 |
|  | dorsal striatum | globus pallidus; ventromedial putamen; dorsal caudate; dorsolateral putamen | 221 - 222; 225 - 226; 227 – 230 |

|  | **region** | **subregions** | **BNA labels** |
| --- | --- | --- | --- |
| ***univariate***  ***prototype/exemplar representations*** | posterior occipital cortex | caudal lingual gyrus, rostral & caudal cuneus; occipital polar cortex | 189 - 194; 203 - 204 |
|  | lateral occipital cortex | middle & inferior OccG | 199 - 200; 205 - 206 |
|  | fusiform gyrus (FUS) | rostroventral, medioventral & lateroventral FUS | 103 - 108 |
| ***multivariate***  ***prototype/exemplar representations***  ***(exporatory analyses)*** | **intraparietal sulcus (IPS)** | **rostrodorsal inferior parietal lobe; rostral, caudal & lateral superior parietal lobe** | **137 – 140; 125 - 130** |

BNA: Brainnetome Atlas. Uneven BNA labels stand for regions in the left hemisphere, even BNA labels stand for regions in the right hemisphere.

Supplementary Methods, Table 1, continued. Anatomical regions of interest.

## Formula of linear models and linear mixed models for data from the training phase

### ***Data from first 8 training rounds***

(S1) accuracy ~ AQ group * category membership * round + (1 | subject id)

(S2) accuracy for category members ~ AQ group * distortion level * round + (1 | subject id)

(S3) accuracy ~ ‘attention to detail’ scores * category membership +

‘attentional switch’ scores * category membership +

‘social interaction’ scores * category membership +

‘communication’ scores * category membership +

‘imagination’ scores * category membership + (1 | subject id)

### ***Data from all training rounds***

(S4) round where performance criterion was reached ~ AQ group

(S5) accuracy ~ AQ group * category membership + (1 | subject id)

### ***Parameter estimates from mixture model estimated on data from first 8 training rounds***

(S6) parameter estimate ~ AQ group * round + (1 | subject id)

## Formula of linear models and linear mixed models for data from the transfer phase

### ***Data from all 8 transfer rounds***

(S7) accuracy ~ AQ group * category membership * round + (1 | subject id)

(S8) accuracy for novel category members ~ AQ group * distortion level + (1 | subject id)

(S9) accuracy for items distorted by level 7 ~ AQ group * category membership * item novelty + (1 | subject id)

### ***Parameter estimates from mixture model estimated on data from all transfer rounds***

(S10) parameter estimate ~ AQ group
