# Supplementary material for: Linking Subclinical Autistic Traits and Perceptual Category Learning
Source: Eur J Neurosci. 2025 Feb 17;61(4):e70000. doi: 10.1111/ejn.70000 (PMC11831246; doi:10.1111/ejn.70000)
Supplement: Supplementary file 2 — Supporting Information S2 Supplementary Results. [file EJN-61-0-s001.docx]

Linking subclinical autistic traits and perceptual category learning

## Supplementary Results

Claire Warren, Rebekka Baumert, Kira Diermann, Daniel Schöttle, Janine Bayer

## AQ group characteristics

Supplementary Results, Table 1. AQ group characteristics.

|  | **AQ_low_** | | **AQ_high_** | | **Two-Sample *t*-tests** | | | |
| --- | --- | --- | --- | --- | --- | --- | --- | --- |
|  | ***M*** | ***SD*** | ***M*** | ***SD*** | **95 % CI** | | ***t*** | ***p_unc_*** |
| **Age** | 25.81 | 3.71 | 25.24 | 3.74 | -1.34 | 2.48 | .60 | .552 |
| **Years of Education** | 15.0 | 2.20 | 13.72 | 1.73 | .27 | 2.29 | 2.53 | **.014*** |
|  |  |  |  |  |  |  |  |  |
| **Non-verbal Intelligence** | 30.41 | 3.75 | 29.41 | 4.17 | -1.05 | 3.03 | .97 | .334 |
| **Working Memory** | .12 | .12 | .15 | .13 | -.09 | .04 | -.84 | .406 |
|  |  |  |  |  |  |  |  |  |
| **Recognition Memory** |  |  |  |  |  |  |  |  |
| hits - false alarms, 1-back | .81 | .17 | .83 | .23 | -.10 | -.06 | -.56 | .580 |
| hits - false alarms, 2-back | .67 | .19 | .66 | .25 | -.11 | .12 | .13 | .90 |
| source memory, 1-back | .28 | .18 | .33 | .19 | -.15 | .05 | -.98 | .329 |
| source memory, 2-back | .49 | .22 | .58 | .21 | -.20 | .02 | -1.70 | .095 |
|  |  |  |  |  |  |  |  |  |
| **Psychiatric Symptoms** |  |  |  |  |  |  |  |  |
| anxiety | 2.25 | 1.57 | 2.21 | 2.72 | -1.12 | 1.20 | .07 | .941 |
| depression | 1.13 | 1.52 | 2.14 | 3.08 | -2.29 | .26 | -1.60 | .117 |
| somatization | .75 | 1.19 | 1.31 | 1.54 | -1.27 | .15 | -1.58 | .120 |
|  |  |  |  |  |  |  |  |  |
| **Emotional Quotient** | 48.44 | 11.68 | 39.69 | 9.91 | 3.21 | 14.28 | 3.16 | **.002**** |
| **Systemizing Quotient** | 42.39 | 15.55 | 56.17 | 22.96 | -23.98 | -3.58 | -2.72 | **.009**** |

## Subscale analyses on accuracy rates from the training phase


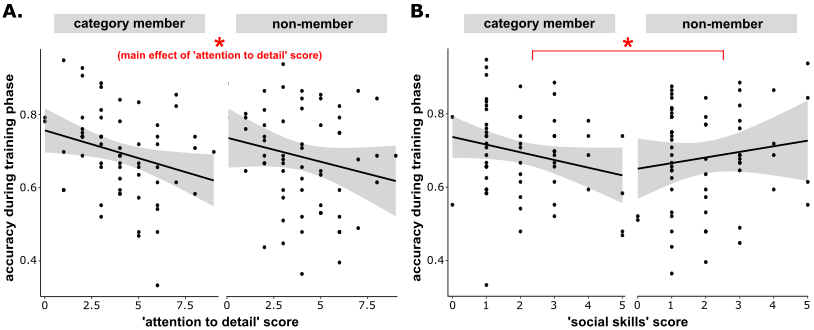


Supplementary Results, Figure 1. Robust regression analyses between accuracy during the training phase and ‘attention to detail’ (A), and to ‘social skills’ scores (B). Grey shaded areas represent 0.95 confidence intervals.

## Subscale analyses on accuracy rates from the transfer phase


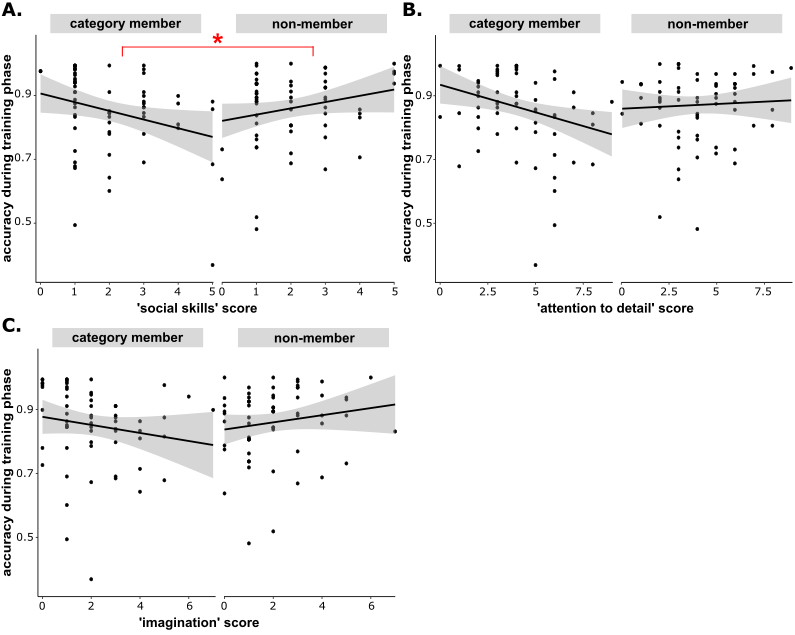


Supplementary Results, Figure 2. Robust regression analyses between accuracy during the transfer phase and ‘attention to detail’ (A), ‘social skills’ (B), and ‘imagination’ scores (C). Grey shaded areas represent 0.95 confidence intervals.

## Main effects during the training phase within regions of interest

Supplementary Results, Table 2

|  |  | | | | |  | | | | **MNI coordinates** | | |  |  | | |  |  |
| --- | --- | --- | --- | --- | --- | --- | --- | --- | --- | --- | --- | --- | --- | --- | --- | --- | --- | --- |
|  | **region** | | | | | **hemisphere** | | | | ***x*** | ***y*** | ***z*** | ***t*** | ***Z*** | ***p*_unc_** | ***p*_cor_** |  | ***k*** |
|  |  | | | | |  | | | |  |  |  |  |  |  |  |  |  |
| ***stimulus onsets > baseline: ROIs typically associated to visually processing*** | | | | | | | | | | | | |  |  |  |  |  |  |
|  | medioventral fusiform gyrus | | | | | L | | | | -28 | -66 | -10 | 17.07 | 10.22 | <.001 | <.001 |  | 2109 |
|  |  |  |  |  |  | R | | | | 30 | -64 | -10 | 15.08 | 9.62 | <.001 | <.001 |  | 1980 |
|  | rostroventral fusiform gyrus | | | | | L | | | | -34 | -10 | -36 | 6.96 | 5.92 | <.001 | <.001 |  | 42 |
|  |  | | | | |  | | | |  |  |  |  |  |  |  |  |  |
| ***stimulus onsets < baseline: ROIs typically associated to visually processing*** | | | | | | | | | | | | |  |  |  |  |  |  |
|  | caudal cuneus | | | | | L/R | | | | 4 | -84 | 36 | 11.28 | 8.20 | <.001 | <.001 |  | 436 |
|  | caudoventral inferior temporal gyrus | | | | | R | | | | 50 | -42 | -22 | 7.98 | 6.55 | <.001 | <.001 |  | 345 |
|  | caudolateral inferior temporal gyrus | | | | | L | | | | -60 | -32 | -12 | 7.53 | 6.28 | <.001 | <.001 |  | 447 |
|  |  | | | | |  | | | |  |  |  |  |  |  |  |  |  |
| ***non-member > member: ROIs typically associated to visually processing*** | | | | | | | | | | | | |  |  |  |  |  |  |
|  | caudal cuneus | | | | | R | | | | 18 | -94 | 2 | 9.52 | 7.38 | <.001 | <.001 |  | 380 |
|  | caudal lingual gyrus | | | | | L | | | | -16 | -86 | -6 | 6.35 | 5.52 | <.001 | .004 |  | 94 |
|  |  | | | | |  | | | |  |  |  |  |  |  |  |  |  |
| ***stimulus onsets > baseline: ROIs typically associated to perceptual decision making*** | | | | | | | | | | | | | |  |  |  |  |  |
|  | medial superior frontal gyrus | | | | | L/R | | | | -4 | 10 | 46 | 21.22 | 11.24 | <.001 | <.001 |  | 759 |
|  | ventrolateral middle frontal gyrus | | | | | L | | | | -26 | -4 | 52 | 18.17 | 10.51 | <.001 | <.001 |  | 270 |
|  |  |  |  |  |  | R | | | | 26 | -2 | 50 | 14.85 | 9.54 | <.001 | <.001 |  | 144 |
|  | ventral middle frontal gyrus | | | | | R | | | | 42 | 32 | 18 | 7.11 | 6.02 | <.001 | <.001 |  | 84 |
|  | dorsal middle frontal gyrus | | | | | R | | | | 32 | 38 | 30 | 8.97 | 7.09 | <.001 | <.001 |  | 149 |
|  | medial inferior frontal junction | | | | | L | | | | -38 | 2 | 28 | 11.67 | 8.37 | <.001 | <.001 |  | 656 |
|  |  |  |  |  |  | R | | | | 38 | 6 | 28 | 8.07 | 6.60 | <.001 | <.001 |  | 33 |
|  | dorsal anterior insula | | | | | R | | | | 32 | 24 | 2 | 15.69 | 9.81 | <.001 | <.001 |  | 370 |
|  | dorsolateral putamen | | | | | L | | | | -30 | -20 | 2 | 15.38 | 9.71 | <.001 | <.001 |  | 322 |
|  | caudal posterior cingulate gyrus | | | | | L | | | | -4 | -10 | 46 | 7.15 | 6.05 | <.001 | <.001 |  | 11 |
|  |  | | | | |  | | | |  |  |  |  |  |  |  |  |  |
| ***stimulus onsets < baseline: ROIs typically associated to perceptual decision making*** | | | | | | | | | | | | | |  |  |  |  |  |
|  | lateral medial superior frontal gyrus | | | | | R | | | | 12 | 24 | 58 | 7.08 | 6.00 | <.001 | <.001 |  | 63 |
|  | opercular inferior frontal gyrus | | | | | L | | | | -46 | 26 | -4 | 10.62 | 7.91 | <.001 | <.001 |  | 72 |
|  |  |  |  |  |  | R | | | | 48 | 32 | -4 | 7.98 | 6.54 | <.001 | <.001 |  | 20 |
|  | lateral inferior frontal junction | | | | | L | | | | -44 | 12 | 52 | 8.85 | 7.03 | <.001 | <.001 |  | 361 |
|  |  |  |  |  |  | R | | | | 48 | 16 | 46 | 6.65 | 5.72 | <.001 | <.001 |  | 476 |
|  | dorsal middle frontal gyrus | | | | | L | | | | -16 | 50 | 28 | 7.51 | 6.27 | <.001 | <.001 |  | 81 |
|  | area 46 of the middle frontal gyrus | | | | | R | | | | 18 | 58 | 18 | 6.61 | 5.69 | <.001 | <.001 |  | 73 |
|  | lateral middle frontal gyrus | | | | | L | | | | -12 | 60 | -4 | 6.10 | 5.35 | <.001 | <.001 |  | 28 |
|  | ventromedial putamen | | | | | L | | | | -32 | -14 | -8 | 6.90 | 5.88 | <.001 | <.001 |  | 13 |
|  |  |  |  |  |  | R | | | | 28 | 4 | -2 | 6.29 | 5.48 | <.001 | <.001 |  | 21 |
|  | dorsal posterior cingulate gyrus | | | | | L/R | | | | -10 | -44 | 34 | 14.37 | 9.39 | <.001 | <.001 |  | 1008 |
|  | |  |  |  |  | |  |  |  |  |  |  |  |  |  |  |  |  |
| ***positive > negative feedback: ROIs typically associated to feedback processing*** | | | | | | | | | | | | |  |  |  |  |  |  |
|  | dorsolateral superior frontal gyrus | | | | | L | | | | -20 | 32 | 48 | 5.59 | 4.99 | <.001 | .005 |  | 39 |
|  | medial superior frontal gyrus | | | | | L/R | | | | 6 | -12 | 50 | 6.17 | 5.40 | <.001 | <.001 |  | 13 |
|  | ventral caudate, ventral and dorsal striatum | | | | | L | | | | -10 | 10 | -2 | 12.41 | 8.67 | <.001 | <.001 |  | 603 |
|  |  |  |  |  |  | R | | | | 10 | 10 | -4 | 13.80 | 9.19 | <.001 | <.001 |  | 731 |
|  | ventral posterior cingulate gyrus | | | | | L/R | | | | -4 | -52 | 18 | 5.91 | 5.21 | <.001 | .002 |  | 33 |
|  | subgenual anterior cingulate gyrus | | | | | L/R | | | | 0 | 44 | -10 | 8.37 | 6.77 | <.001 | <.001 |  | 152 |
|  |  | | | | |  | | | |  |  |  |  |  |  |  |  |  |
| ***negative > positive feedback: ROIs typically associated to feedback processing*** | | | | | | | | | | | | |  |  |  |  |  |  |
|  | medial superior frontal gyrus | | | | | L/R | | | | 0 | 16 | 60 | 9.84 | 7.54 | <.001 | <.001 |  | 796 |
|  |  |  |  |  |  | R | | | | 8 | 26 | 34 | 7.38 | 6.19 | <.001 | <.001 |  | 328 |
|  | dorsal middle frontal gyrus | | | | | L | | | | -24 | 44 | 22 | 9.09 | 7.16 | <.001 | <.001 |  | 208 |
|  |  |  |  |  |  | R | | | | 22 | 48 | 30 | 6.08 | 5.33 | <.001 | .001 |  | 37 |
|  | rostral inferior frontal gyrus | | | | | R | | | | 52 | 24 | -4 | 7.24 | 6.10 | <.001 | <.001 |  | 176 |
|  |  | | | | |  | | | |  |  |  |  |  |  |  |  |  |
| ***dissimilarity to prototype/exemplars*** | | | | | | | | | |  |  |  |  |  |  |  |  |  |
|  | lateroventral fusiform gyrus | | | | | R | | | | 46 | -48 | -16 | 6.43 | 5.57 | <.001 | <.001 |  | 110 |
|  | medioventral fusiform gyrus | | | | | L | | | | 36 | -66 | -12 | 5.4 | 4.85 | <.001 | 0.004 |  | 23 |
|  | occipito polar cortex | | | | | L | | | | -20 | -92 | 0 | 4.99 | 4.54 | <.001 | 0.015 |  | 4 |
|  | caudal cuneus | | | | | L | | | | -12 | -92 | -2 | 4.73 | 4.34 | <.001 | .033 |  | 3 |

## Main effects during the transfer phase within regions of interest

Supplementary Results, Table 3

|  |  |  | **MNI coordinates** | | |  |  | | |  |  |
| --- | --- | --- | --- | --- | --- | --- | --- | --- | --- | --- | --- |
|  | **region** | **hemisphere** | ***x*** | ***y*** | ***z*** | ***t*** | ***Z*** | ***p*_unc_** | ***p*_cor_** |  | ***k*** |
|  |  |  |  |  |  |  |  |  |  |  |  |
| ***stimulus onsets > baseline: ROIs typically associated to visual processing*** | | | | | |  |  |  |  |  |  |
|  | medioventral fusiform gyrus | L | -30 | -72 | -10 | 20.84 |  | <.001 | <.001 |  | 3060 |
|  |  | R | 30 | -68 | -10 | 18.00 |  | <.001 | <.001 |  | 2622 |
|  | rostroventral fusiform gyrus | L | -32 | -12 | -38 | 7.48 | 6.25 | <.001 | <.001 |  | 84 |
|  |  | R | 34 | -14 | -38 | 8.95 | 7.09 | <.001 | <.001 |  | 188 |
|  |  |  |  |  |  |  |  |  |  |  |  |
|  |  |  |  |  |  |  |  |  |  |  |  |
| ***stimulus onsets < baseline: ROIs typically associated to visual processing*** | | | | | |  |  |  |  |  |  |
|  | rostral cuneus | L/R | -4 | -84 | 26 | 12.01 |  | <.001 | <.001 |  | 670 |
|  | caudolateral inferior temporal gyrus | L | -54 | -42 | -8 | 9.80 | 7.52 | <.001 | <.001 |  | 814 |
|  | intermediate lateral inferior temporal gyrus | R | 58 | -10 | -28 | 9.54 | 7.39 | <.001 | <.001 |  | 614 |
|  | rostral temporal gyrus | L | -48 | 0 | -40 | 5.43 | 4.87 | <.001 | .005 |  | 31 |
|  |  |  |  |  |  |  |  |  |  |  |  |
| ***non-member > member: ROIs typically associated to visual processing*** | | | | | |  |  |  |  |  |  |
|  | caudal lingual gyrus and occipital polar cortex | R | 18 | -94 | 0 | 8.7 | 6.95 | <.001 | <.001 |  | 242 |
|  | occipital polar cortex | L | -22 | -92 | -4 | 7.06 | 5.99 | <.001 | <.001 |  | 186 |
|  |  |  |  |  |  |  |  |  |  |  |  |
| ***stimulus onsets > baseline: ROIs typically associated to perceptual decision making*** | | | | | | |  |  |  |  |  |
|  | medial superior frontal gyrus | L/R | -4 | 6 | 48 | 19.99 |  | <.001 | <.001 |  | 865 |
|  | anterior insula | L | -28 | 22 | 4 | 19.65 |  | <.001 | <.001 |  | 408 |
|  |  | R | 32 | 22 | 0 | 19.15 |  | <.001 | <.001 |  | 604 |
|  | ventrolateral middle frontal gyrus | L | -28 | -6 | 52 | 16.00 |  | <.001 | <.001 |  | 191 |
|  |  | R | 28 | -2 | 52 | 13.70 |  | <.001 | <.001 |  | 109 |
|  | inferior frontal junction | L | -38 | 2 | 28 | 11.24 |  | <.001 | <.001 |  | 418 |
|  |  | R | 40 | 10 | 30 | 9.35 | 7.29 | <.001 | <.001 |  | 109 |
|  | latero-dorsal middle frontal gyrus | R | 32 | 40 | 26 | 10.12 | 7.67 | <.001 | <.001 |  | 540 |
|  | inferior dorsal posterior cingulate gyrus | L/R | -4 | -30 | 26 | 9.88 | 7.56 | <.001 | <.001 |  | 199 |
|  | caudal posterior cingulate gyrus | L/R | -6 | -10 | 46 | 7.56 | 6.30 | <.001 | <.001 |  | 12 |
|  |  |  |  |  |  |  |  |  |  |  |  |
| ***stimulus onsets < baseline: ROIs typically associated to perceptual decision making*** | | | | | | |  |  |  |  |  |
|  | ventromedial putamen | R | -24 | 4 | -10 | 15.82 |  | <.001 | <.001 |  | 154 |
|  |  | R | 26 | 4 | -8 | 13.67 |  | <.001 | <.001 |  | 89 |
|  | superior dorsal posterior cingulate gyrus | L | -10 | -46 | 34 | 12.65 |  | <.001 | <.001 |  | 822 |
|  | opercular inferior frontal gyrus | L | -48 | 28 | 0 | 11.25 |  | <.001 | <.001 |  | 83 |
|  | ventrolateral middle frontal gyrus | L | -36 | 24 | 42 | 11.21 |  | <.001 | <.001 |  | 797 |
|  |  | R | 34 | 24 | 44 | 9.35 | 7.29 | <.001 | <.001 |  | 795 |
|  | medial superior frontal gyrus | L | -10 | 20 | 58 | 8.29 | 6.73 | <.001 | <.001 |  | 36 |
|  | ventral posterior cingulate gyrus | L | -16 | -50 | 6 | 8.12 | 6.63 | <.001 | <.001 |  | 93 |
|  |  | R | 14 | -46 | 4 | 6.00 | 5.28 | <.001 | <.001 |  | 13 |
|  | medio-dorsal middle frontal gyrus | L | -16 | 44 | 32 | 7.93 | 6.52 | <.001 | <.001 |  | 67 |
|  | medial superior frontal gyrus | R | 16 | 22 | 60 | 6.16 | 5.39 | <.001 | <.001 |  | 18 |
|  | middle frontal gyrus area 46 | R | 18 | 56 | 18 | 5.73 | 5.09 | <.001 | .002 |  | 9 |
|  |  |  |  |  |  |  |  |  |  |  |  |
| ***dissimilarity to prototype*** | |  |  |  |  |  |  |  |  |  |  |
|  | lateral inferior occipital gyrus | R | 34 | -86 | -8 | 5.47 | 4.90 | <.001 | .003 |  | 33 |
|  |  | R | 44 | -72 | -8 | 5.22 | 4.71 | <.001 | .007 |  | 15 |
|  | lateroventral fusiform gyrus | L | -42 | -58 | -16 | 4.57 | 4.21 | <.001 | .050 |  | 1 |
|  |  | R | 42 | -50 | -16 | 5.23 | 4.72 | <.001 | .007 |  | 28 |
|  |  | R | 36 | -38 | -18 | 4.92 | 4.49 | <.001 | .017 |  | 10 |
|  |  | R | 44 | -64 | -12 | 4.81 | 4.40 | <.001 | .025 |  | 4 |
|  |  |  |  |  |  |  |  |  |  |  |  |
|  |  |  |  |  |  |  |  |  |  |  |  |
| ***similarity to exemplars*** | |  |  |  |  |  |  |  |  |  |  |
|  | occipito polar cortex | L | -30 | -96 | -10 | 6.69 | 5.75 | <.001 | <.001 |  | 136 |
|  |  | R | 30 | -94 | -2 | 7.55 | 6.29 | <.001 | <.001 |  | 411 |
|  | posterior inferior occipital gyrus | R | 48 | -80 | -12 | 5.32 | 4.79 | <.001 | .005 |  | 9 |
|  | lateroventral fusiform gyrus | L | -38 | -58 | -16 | 4.72 | 4.33 | <.001 | .032 |  | 5 |
|  |  |  |  |  |  |  |  |  |  |  |  |
| ***dissimilarity to exemplars*** | |  |  |  |  |  |  |  |  |  |  |
|  | medioventral occipital cortex | L | -16 | -92 | -4 | 8.1 | 6.61 | <.001 | <.001 |  | 214 |
|  |  | R | 12 | -88 | -6 | 6.23 | 5.44 | <.001 | <.001 |  | 65 |

## Analyses on baseline activity during the training phase

To investigate whether group effects occurring for contrasting activity related to stimulus onsets against activity related to fixation cross onsets could be accounted by differences in explicit baseline activity, first-level regressors containing fixation cross onsets were contrasted against implicit baseline. Individual contrast images were submitted to two-sample *t*-tests. Next, contrast estimates were extracted from ROIs in which group effects were found and further analyzed with robust linear mixed models. Regarding group by training interactions detected in ROIs typically associated to visual processing, neither baseline activity in the middle occipital gyrus (*t*(885) = -.724, *p*_unc_ =.469, *p*_cor_ = .334, 95%CI [ -.187, .086]) and nor in the rostral cuneus (*t*(885) = 1.38, *p*_unc_ =.170, *p*_cor_ = .938, 95%CI [ -.038, .223]) accounted for the observed effects. Likewise, analyses did not suggest that group by hemisphere interactions in the ventrolateral midfrontal gyrus area 8 (*t*(885) = -1.72, *p*_unc_ =.085, *p*_cor_= .255, 95%CI [ -.107, .007]) and area 6 (*t*(885) = -.46, *p*_unc_ =.642, *p*_cor_ > .999, 95%CI [ -.072, .045]) can be accounted by such interactions in baseline activity. Moreover, there was no indication that the group by round interaction observed in the posterior cingulum can be accounted by such an interaction in baseline activity 8 (*t*(885) = -1.40, *p*_unc_ =.162, *p*_cor_ > .999, 95%CI [ -.263, .044]).

## Analyses on baseline activity during the transfer phase

As for the training phase, first-level regressors containing fixation cross onsets were contrasted against implicit baseline. Again, contrast estimates were extracted from ROIs in which significant group effects were found in analyses on the contrast between activity related to stimulus onsets against activity related to fixation cross onsets (see section XX, main manuscript). Analyses suggested that group differences in baseline activity could have contributed to group by hemisphere interactions in the ventrolateral middle frontal gyrus (*t*(59) = -2.70, *p*_unc_ =.009, *p*_cor_ = .027, 95%CI [ -.275, -.055]), the opercular inferior frontal gyrus (*t*(59) = -2.92, *p*_unc_ =.008, *p*_cor_ = .025, 95%CI [ -.280, -.046]) and the anterior insula (*t*(59) = -2.73, *p*_unc_ =.005, *p*_cor_ = .015, 95%CI [ -.229, -.045]).

## Model fits for training phase data

Supplementary Results, Table 4. Delta DIC’s for data from the training phase.

| **Model** | ***M_deltaDIC_*** |
| --- | --- |
| MIX-2c-γ | 39.36 |
| MIX-γ | 39.05 |
| EX-γ | 37.22 |
| EX | 32.21 |
| MIX | 30.85 |
| MIX-2c | 30.32 |
| PROTO | 26.52 |

## Model fits for transfer phase data

Supplementary Results, Table 5. Delta DIC’s for data from the transfer phase.

| **Model** | ***M_deltaDIC_*** |
| --- | --- |
| MIX | 52.2 |
| MIX-2c | 51.51 |
| EX | 49.1 |
| EX-γ | 47.94 |
| PROTO | 45.88 |

## Exploratory ROI-based analyses within the IPS


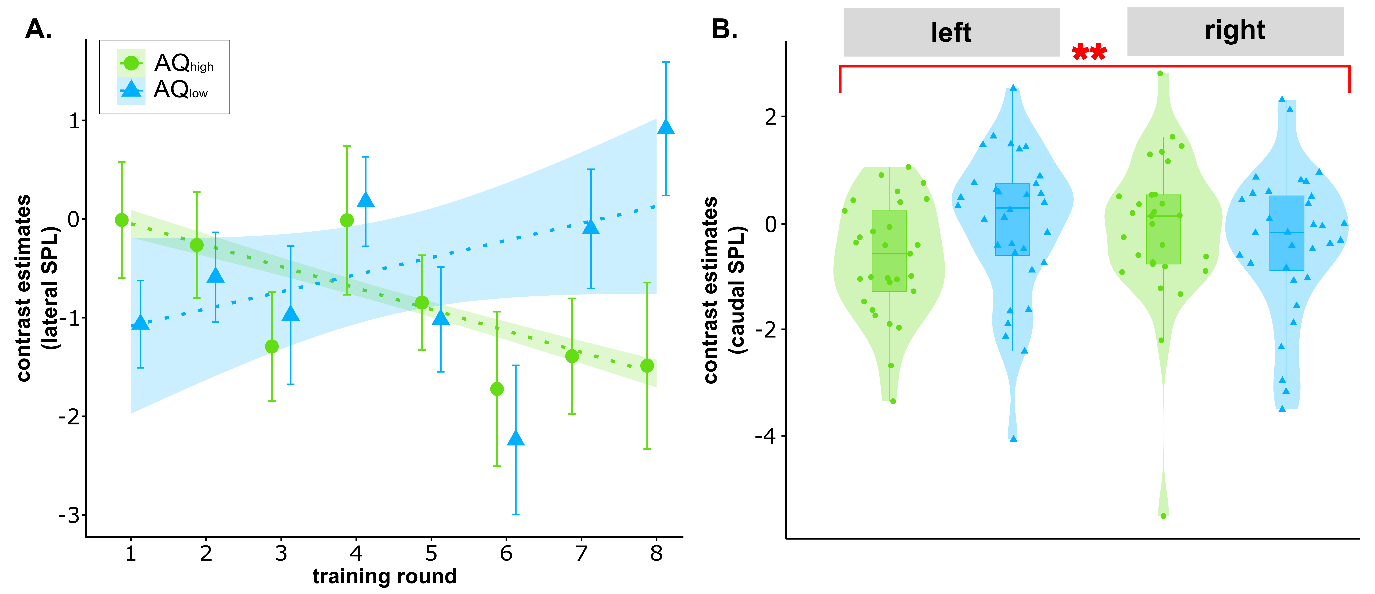


Supplementary Results, Figure 3. Exploratory analyses within the intraparietal sulcus (IPS), covering sections of the superior parietal lobe (SPL) and the inferior parietal lobe. A. Contrast estimates during the training phase from the lateral SPL for AQ_high_ and AQ_low_, indicating increasingly negative prototype/exemplar representations across training rounds in AQ_high_ and an opposite pattern in the AQ_low_ group. B. Contrast estimates during the training phase from the caudal SPL for AQ_high_ and AQ_low_, indicating that only the AQ_high_ group exhibited a pronounced negative correlation between prototype similarity and activity in the left hemisphere. Significant interactions are highlighted with red asterisks (** p < .01).
